# Integrated multiomics profiling elucidates the spatiotemporal metabolic dynamics and regulatory networks of the bioactive components of *Trichosanthes kirilowii*

**DOI:** 10.3389/fpls.2026.1735703

**Published:** 2026-02-17

**Authors:** Haiyun Gao, Xiaoai Li, Chu Wang, Yao Li, Tianrui Liu, Nana Chang, Yang Xu, Ye Wang, Yan Ren, Gao Zhou, Wei Gao, Ying Zeng, Huan Zhao, Hui Li

**Affiliations:** 1 Key Laboratory of Sustainable Utilization of Traditional Chinese Medicine Resources in Jiangxi Province, Institute of Traditional Chinese Medicine Health Industry, China Academy of Chinese Medical Sciences, Nanchang, China; 2 Center for Research on Characteristic Resources of Big Health, Jiangxi Health Industry Institute of Traditional Chinese Medicine, Nanchang, China; 3 School of Traditional Chinese Medicine, Capital Medical University, Beijing, China; 4 Institute of Chinese Materia Medica, China Academy of Chinese Medical Sciences, Beijing, China

**Keywords:** bioactive compounds, medicinal and edible plant, metabolic regulation, multiomics integration, transcription factor, *Trichosanthes kirilowii*

## Abstract

**Introduction:**

*Trichosanthes kirilowii* Maxim. is an important medicinal and edible plant, with its roots, fruits, pericarp, and seeds extensively utilized in traditional medicine and increasingly incorporated into functional foods and health products due to their distinct bioactive constituents. However, a comprehensive understanding of the spatiotemporal dynamics and regulatory mechanisms underlying the biosynthesis of these valuable compounds is lacking, limiting the scientific basis for traditional use, targeted quality improvement, and value-added utilization of this plant.

**Methods:**

An integrated multiomics strategy (LC–MS/MS metabolomics and Illumina transcriptomics) was employed to elucidate the metabolic and transcriptional landscapes across three pivotal fruit ripening stages (initial, color-changing, and mature) and four tissues (roots, pericarp, fruits, and seeds). Weighted gene coexpression network analysis (WGCNA) was used to identify core modules associated with medicinal compound biosynthesis and hub transcription factors.

**Results:**

A total of 1,558 metabolites were identified via metabolomic profiling, which revealed pronounced tissue-specific accumulation: fruits and pericarps were enriched in amino acids, flavonoids, and organic acids, seeds accumulated terpenoids, flavonoids, and fatty acids, roots served as the predominant reservoir for pharmacologically active terpenoids, such as the antitumor cucurbitacin B. Transcriptome analysis revealed tissue- and stage-specific expression patterns of genes involved in terpenoid, phenylpropanoid, and flavonoid biosynthetic pathways, which were strongly correlated with metabolite abundance. WGCNA identified three core modules (MEturquoise, MEblack, and MEbrown) and pinpointed the transcription factor *Tk_ERF4* as a putative regulator orchestrating cross-talk between these metabolic pathways—supported by consistent co-expression patterns with key pathway genes and conservation of *Tk_ERF4* function in medicinal plants.

**Discussion:**

Collectively, our findings provide a comprehensive molecular blueprint for the spatiotemporal biosynthesis of medicinal compounds in *T. kirilowii*, deciphering the scientific basis for traditional organ-specific use, establishing foundations for genetic enhancement and quality control, and offering scientific guidance for precision horticultural practices. Beyond *T. kirilowii*, this work provides a valuable multiomics reference for medicinal-edible plant research and serves as a methodological paradigm for bridging traditional knowledge with modern bioscience.

## 1 Introduction

*Trichosanthes kirilowii* Maxim. (Cucurbitaceae), known as Gualou in Chinese, represents a quintessential example of a medicinal and edible homologous species in China. It has distinct tissue-specific applications in traditional Chinese medicine (TCM) and holds significant potential in the modern functional food and nutraceutical industries. The fruit (*Trichosanthis Fructus*) is primarily used to clear heat and resolve phlegm, especially for relieving pulmonary heat-induced cough in respiratory care, while the root (*Trichosanthis Radix/Tianhuafen*) addresses wasting-thirst syndrome (diabetes management). Concurrently, the seeds (*Trichosanthis Semen*) have laxative properties, and their oil serves as a functional food component for cardiovascular health (Hou et al., 2020; Wang et al., 2024). The diverse applications of its organs underscore the plant’s economic and health value. Modern pharmacology confirms these effects stem from tissue-specific bioactive compounds (e.g., triterpenoids, flavonoids, alkaloids) (Yu et al., 2018). Notably, fruit-abundant cucurbitacin B inhibits the STAT3 pathway to exert antitumor effects (Nie et al., 2024; Dai et al., 2023), root-specific trichosanthin has immunomodulatory and anti-HIV properties (Shaw et al., 2005), and seed lipids (35% of seed weight, over 60% linoleic acid) offer nutritional and hypolipidemic benefits (Chen et al., 2024; Xu Y, et al., 2022). Bioactive accumulation is developmentally regulated—for instance, 3,29-dibenzoyl rarounitriol accumulates during fruit ripening and peaks at maturity (Liu et al., 2014), indicating spatiotemporal regulation that determines raw material quality and suitability.

Despite these well-documented activities and the successful use of transcriptomic–metabolomic integration to decode secondary metabolism in medicinal plants (e.g., *Panax ginseng*, *Astragalus membranaceus*) (Liu et al., 2023; Guan et al., 2025), two critical knowledge gaps impede the scientific guidance for *T. kirilowii* cultivation and quality optimization. First, prior studies focused on single tissues (e.g., fruits, roots) and lacked holistic metabolomic comparisons across all pharmacologically relevant organs (roots, fruits, seeds, and pericarp). This limits systems-level insights into metabolic resource allocation, tissue-specific quality standards, and full exploitation of its medicinal-edible value. Second, the coordinated gene–metabolite regulatory dynamics during fruit maturation—critical for *Trichosanthis Fructus* quality—remain poorly defined. Key drivers, especially transcription factor-mediated regulatory networks controlling bioactive compound flux, are largely unknown. These gaps hamper targeted metabolic manipulation via breeding/cultivation to meet pharmaceutical or functional food needs.

To fill these gaps, we integrated LC–MS/MS (Liquid Chromatography-Tandem Mass Spectrometry) metabolomics and Illumina transcriptomics to decipher metabolic differentiation across four *T. kirilowii* tissues and track pathway reprogramming during three critical fruit developmental stages. We aim to define transcriptional regulatory modules for tissue-specific medicinal compound biosynthesis and identify ripening-associated hub genes and their metabolic interactions. We expect this analysis to deepen understanding of the metabolic basis for traditional organ-specific use and provide actionable insights for genetic improvement, precision cultivation, and quality control—supporting sustainable development of the Gualou industry. Ultimately, these findings provide a theoretical framework for targeted medicinal organ cultivation, science-based harvesting, and metabolic engineering applications.

## 2 Materials and methods

### 2.1 Plant materials

*T. kirilowii* tissue samples were collected from Jinlouzi Agricultural Development Co., Ltd., (Jiujiang, Jiangxi, China, 29°44′N, 116°43′E). The specimens included fruits at three developmental stages (initial fruit (IF), coloration fruit (CF), and mature fruit (MF)) whole fruits including pericarp, n=6 biological replicates per stage) and four distinct tissues: mature fruits (*Trichosanthis Fructus*, MF), roots (*Trichosanthis Radix*, R), pericarp (*Trichosanthis Pericarpium*, P), and seeds (*Trichosanthis Semen*, S) (n=6 biological replicates per tissue). Samples were immediately flash-frozen in liquid nitrogen and stored at −80 °C until further analysis.

### 2.2 Metabolite extraction and UHPLC–MS/MS analysis

Frozen tissues (100 mg) were pulverized in liquid nitrogen using a mortar and pestle. The homogenate was resuspended in prechilled 80% methanol (v/v) and vortexed thoroughly. After 5 min of incubation on ice, the samples were centrifuged at 15,000 × *g* and 4 °C for 20 min. The supernatant was diluted with LC–MS-grade water to a final methanol concentration of 53% (v/v), after which the sample was recentrifuged under identical conditions and filtered through a 0.22-μm nylon membrane. The processed extracts were stored at −80 °C prior to LC–MS/MS analysis.

Untargeted metabolomics was performed by Novogene Biotechnology Co., Ltd. (Beijing, China) using a Vanquish UHPLC system coupled with an Orbitrap Q Exactive™ HF-X mass spectrometer (Thermo Fisher Scientific, Germany). Chromatographic separation was achieved on a Hypersil Gold C18 column (100 × 2.1 mm, 1.9 μm; Thermo Fisher) with gradient elution over 12 min (0.2 mL/min), as follows: 2% B (0.1% formic acid in methanol) → 85% B (3 min) → 100% B (10 min) → re-equilibration at 2% B (10.1–12 min). MS parameters were as follows: ESI ± ionization mode, spray voltage of 3.5 kV, capillary temperature of 320 °C, sheath gas pressure of 35 psi, auxiliary gas flow rate of 10 L/min, S-lens RF level of 60, and gas heater temperature of 350 °C.

### 2.3 Data processing and metabolite identification

The raw data were processed for peak alignment, peak selection, and quantification using Compound Discoverer 3.3 (Thermo Fisher). Key parameters included a mass tolerance of 5 ppm, a signal intensity tolerance of 30%, a minimum intensity threshold, and QC-based peak area normalization. The peak intensities were normalized to the total spectral intensity. Metabolites were annotated using mzCloud (https://www.mzcloud.org/), mzVault, and MassList. To ensure data quality for subsequent biological interpretation, peaks with coefficients of variation (CVs) >30% in the QC samples were excluded. Statistical analyses were performed using R (version 3.4.3), Python (version 2.7.6), and CentOS (release 6.6), with nonnormalized data standardized using the following formula: Relative peak area=Sample raw value × (total sample sum/QC1 sum).

### 2.4 Multivariate data analysis

Metabolites were annotated using the KEGG (https://www.genome.jp/kegg/pathway.html), HMDB (https://hmdb.ca/metabolites) and LIPID MAPS (http://www.lipidmaps.org/) databases. Multivariate statistical analyses including principal component analysis (PCA) and partial least squares discriminant analysis (PLS-DA) were conducted to characterize metabolite diversity. Differentially abundant metabolites (DAMs) were identified on the basis of a variable importance in projection (VIP) >1.0, a *p* value <0.05, and a fold change (FC) ≥1.2 or ≤0.833.

### 2.5 Transcriptomic analysis

Total RNA was extracted using the RNAprep Pure Plant Kit (Polysaccharides & Polyphenolics-rich, Tiangen, Beijing, China). RNA concentration and integrity were determined using an Agilent 2100 bioanalyzer 2100 system (Agilent Technologies, CA, USA). Paired-end sequencing (PE150) was performed on an Illumina NovaSeq X Plus system (Novogene, China). Raw reads were filtered to remove low-quality sequences (Qphred ≤20 in >50% of the read length), joints, and N bases. *De novo* assembly was performed with Trinity (v2.6.6), and completeness was assessed with BUSCO (v3.0.2). Seven databases were used for gene annotation: KEGG, KOG, GO, Pfam, TrEMBL, NR, and Swiss-Prot. Gene expression levels were normalized to fragments per kilobase of transcript per million mapped reads (FPKM). Differential expression analysis was conducted using DESeq2 (1.26.0) software, with screening criteria of |log_2_(fold change)| >1 and a false discovery rate (FDR) <0.05. Selected differentially expressed genes (DEGs) were annotated with the GO and KEGG databases for functional prediction.

### 2.6 qRT–PCR validation

Eight DEGs related to terpene synthesis, four related to flavonoid synthesis, and ten related to phenylpropanoid synthesis were validated by real-time quantitative PCR (qRT–PCR) using *TkGAPDH* as the reference gene. cDNA was synthesized using TransScript^®^ II Uni All-in-one First-Strand cDNA Synthesis SuperMix (TransGen Biotech, Beijing, China). qRT–PCR was performed on the Bio-Rad CFX96 qRT–PCR platform (Bio-Rad Laboratories, Hercules, CA, USA) using PerfectStart Visual Green qPCR SuperMix (TransGen Biotech). Relative expression levels were calculated using the 2^-ΔΔCT^ method (n=3 technical replicates per biological sample). The primer pairs used for the reference genes and all the selected genes all selected genes are listed in **Supplementary Table 1**.

### 2.7 Statistical analysis

All measurements were performed in triplicate (biological replicates: n=6 for metabolomics; n=3 for transcriptomics; technical replicates: n=3 for qRT-PCR). Data analysis was conducted using IBM SPSS Statistics 26. Inter-group differences were analyzed by one-way analysis of variance (ANOVA) followed by Duncan’s multiple comparison test (*p* < 0.05). Histograms were generated using Excel 2010. Bioinformatics analyses and mapping were performed using the NovoMagic platform (https://magic.novogene.com). Gene–metabolite interaction networks were visualized using Cytoscape (v3.10.0).

## 3 Results

### 3.1 Global metabolome landscapes reveal tissue-specific and stage-dependent metabolic diversification

**Figure 1:**
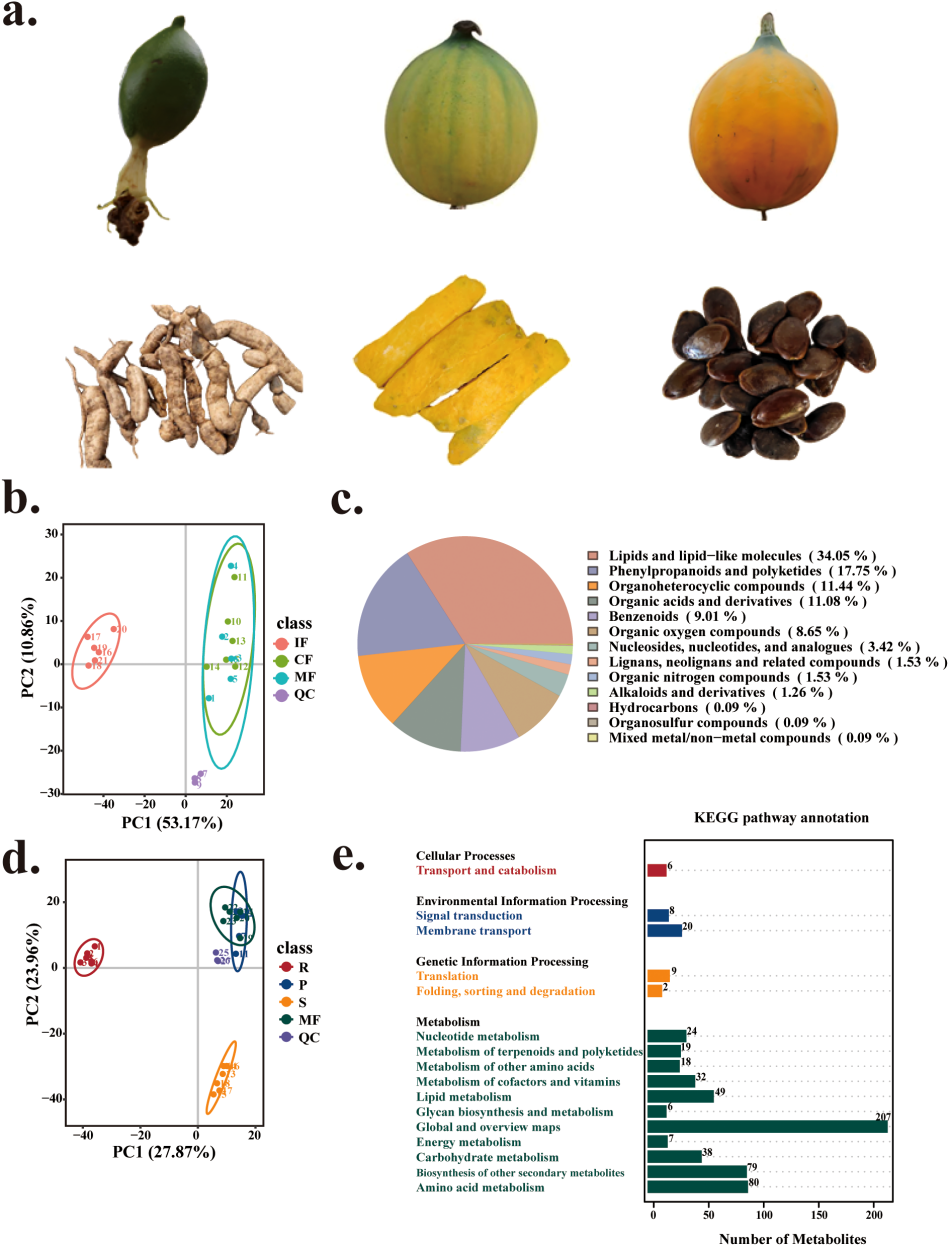
Analysis of tissue-specific and fruit developmental variations in *Trichosanthes kirilowii* and an overview of the metabolite profiles. **(A)** Developmental stages of *Trichosanthes* fruits and sampled tissues. IF (initial fruit), CF (coloration fruit), MF (mature fruit) represent fruit developmental stages; R (root), P (pericarp), S (seed) denote plant tissues. **(B)** Pie chart illustrating the classification of all detected metabolites (n=1,588). **(C)** Principal component analysis (PCA) of the metabolite profiles of distinct tissues. **(D)** PCA of metabolic variations among fruits in different developmental stages. **(E)** KEGG pathway enrichment analysis of identified metabolites.

To systematically elucidate the tissue-specific distribution patterns of secondary metabolites and the regulatory networks governing fruit maturation in *T. kirilowii*, untargeted metabolomics was performed using ultrahigh-performance liquid chromatography coupled with quadrupole time-of-flight mass spectrometry (UHPLC–QTOF–MS) to analyze the global metabolite profiles in fruits (MF), roots (R), pericarp (P), seeds (S), and fruits at three stages of maturation (initial fruit, IF; coloration fruit, CF; and mature fruit, MF) (**Figure 1A**). Pearson correlation coefficients (|r| > 0.95, p < 0.01) confirmed high consistency among biological replicates (n=6 per group; **Supplementary Figure S1**), ensuring data reliability. A total of 1,558 metabolites were identified, with 906 detected in positive ion mode and 652 in negative ion mode. In terms of metabolite classification, lipids and their derivatives constituted the greatest proportion (34.05%), followed by phenylpropanoids/polyketides (17.75%), organoheterocyclic compounds (11.44%), and organic acid derivatives (11.08%) (**Figure 1C**). Principal component analysis (PCA) revealed distinct separation of the root tissue (R) from the pericarp (P) and the seeds (S) along PC1 (29.21% variance) and PC2 (25.58% variance), while partial overlap between mature fruit (MF) and pericarp (P) was observed (**Figure 1B**).

Dynamic analysis of fruit development revealed gradient distributions of initial fruit (IF), coloration fruit (CF), and mature fruit (MF) fruits along PC1 (**Figure 1D**). The metabolic divergence between stage CF and MF was not significant (VIP<1.0, *p*>0.05), with 93.4% of differential metabolites (DAMs) identified between IF and CF, suggesting secondary metabolic reprogramming predominantly occurs in early development. KEGG functional annotation revealed significant enrichment of differentially abundant metabolites in pathways related to secondary metabolite biosynthesis (ko01110), amino acid metabolism (ko01230), lipid metabolism (ko00564), and carbohydrate metabolism (ko00520) (**Figure 1E**). The dynamic changes and tissue-specific accumulation of these metabolites across fruit maturation stages was further analyzed in detail below.

### 3.2 Tissue-specific accumulation patterns of primary and secondary metabolites underlie different bioactivities

#### 3.2.1 Primary metabolites

**Figure 2:**
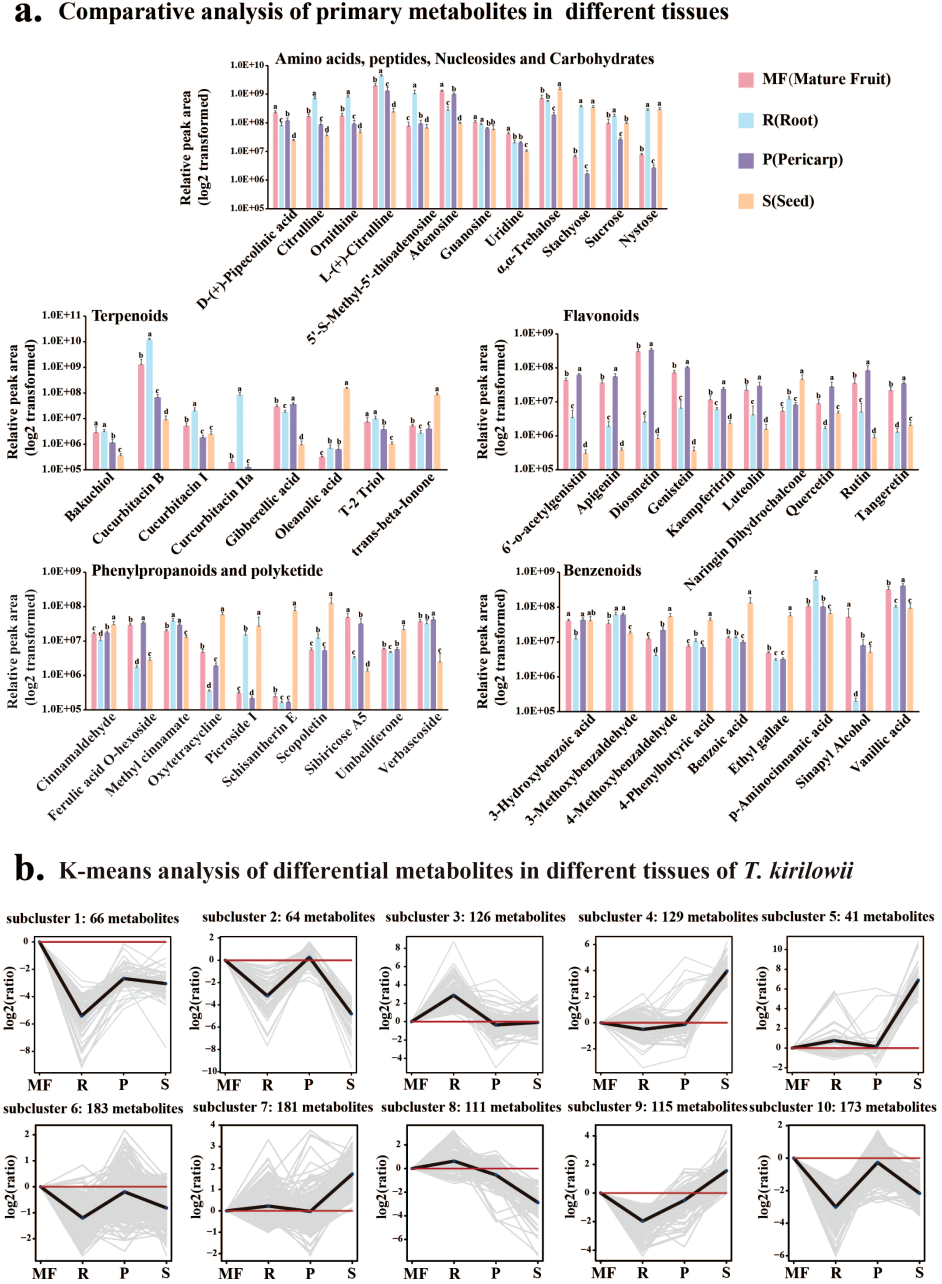
Tissue-specific accumulation of primary and secondary metabolites. **(A)** Comparative analysis of primary (Amino acids, peptides, Nucleosides and Carbohydrates) and secondary (terpenoids, flavonoids, phenylpropanoids and polyketides, benzenoids) metabolites across tissues. Y-axis: Relative peak area (log2 transformed). Different lowercase letters indicate significant differences at *p*<0.05 (Duncan’s multiple comparison; n=6 biological replicates). **(B)** K-means clustering of 1,189 differentially abundant metabolites (DAMs) across tissues, partitioned into 10 clusters with distinct accumulation trends. Clusters 6/7/10 (pericarp/fruit-enriched), Clusters 3/8 (root-enriched), Clusters 4/5/7/9 (seed-enriched).

In this study, 85 sugars and derivatives were identified. The soluble sugars detected in *T. kirilowii* included α,α-trehalose, stachyose, sucrose, nystose, D-raffinose, and α-lactose. Among these, α,α-trehalose, stachyose, nystose, and sucrose were abundant in the roots (R) and seeds (S) but present in relatively low levels in the pericarp (P). α,α-trehalose was the most abundant sugar derivative across all tissues, with the highest relative content in seeds (1.5×10^9^, n=6; **Figure 2A**).

Thirty-eight nucleotides and derivatives were identified (**Supplementary Table S2**), with major components including adenosine, 5’-S-methyl-5’-thioadenosine, guanosine, and uridine. Adenosine was enriched in the fruits (MF) and pericarp (P), whereas 5’-S-methyl-5’-thioadenosine was predominant in the roots (R). Adenosine constituted the most abundant nucleotide across all the tissues. Eighty-nine amino acids and derivatives were identified (**Supplementary Table S2**), with major components including D-(-)-glutamine, L-lysine, D-(+)-pipecolic acid, citrulline, ornithine, and L-(+)-citrulline. L-(+)-Citrulline showed the greatest accumulation across all tissues (**Figure 2A**).

#### 3.2.2 Secondary metabolites

One hundred forty-six terpenoids were identified (**Supplementary Table S2**), encompassing monoterpenes, diterpenes, sesquiterpenes, and triterpenes. Triterpenes dominated this class, primarily including cucurbitacin B, oleanolic acid, ursolic acid, cucurbitacin I, and cucurbitacin IIa. Tissue-specific accumulation of secondary metabolites was evident: cucurbitacin B was significantly enriched in R (1.23×10^10^, n=6) and IF (1.17×10^10^, n=6), with relative content 9.0-1,370.1 folds higher than that in other tissues (*p* < 0.05, Duncan’s multiple comparison); while oleanolic and ursolic acids predominantly accumulated in the seeds (**Figure 2A**). Monoterpenes were dominated by bakuchiol and (-)-camphor, while diterpenes included euphorbia factor L1 and gibberellic acid; T-2 triol and trans-*β*-ionone were highly abundant in S (**Figure 2A**).

Ninety-five flavonoids were identified (**Supplementary Table S2**), categorized as flavans (8), flavones (9), flavonoid glycosides (52), isoflav-2-enes (4), isoflavonoid C-glycosides (1), isoflavonoid O-glycosides (3), O-methylated flavonoids (17), and rotenoids (1). High-abundance flavonoids included kaempferitrin, tangeretin, naringin dihydrochalcone, quercetin, 6’-O-acetylgenistin, apigenin, genistein, luteolin, rutin, and diosmetin. These compounds were predominantly enriched in MF and P (diosmetin showed markedly higher levels), while naringin dihydrochalcone was notably abundant in seeds (**Figure 2A**).

One hundred two phenylpropanoids and polyketides were classified, with major components including caffeic acid, trans-cinnamaldehyde, trans-cinnamic acid, cinnamaldehyde, schisantherin E, sibiricose A5, methyl cinnamate, verbascoside, and scopoletin. Scopoletin, schisantherin E, and cinnamaldehyde were enriched in seeds, whereas sibiricose A5, methyl cinnamate, and verbascoside accumulated primarily in fruits and pericarps (**Figure 2A**). One hundred phenolic derivatives were identified, including 30 phenols such as 3-hydroxybenzoic acid, benzoic acid, 3-methoxybenzaldehyde, *p*-aminocinnamic acid, and vanillic acid. Vanillic acid was highly enriched in fruits and pericarps, while *p*-aminocinnamic acid predominated in the roots (**Figure 2A**).

K-means clustering was applied to 1,189 DAMs, partitioning them into 10 clusters with distinct variation trends (**Figure 2B**). Clusters 6, 7, and 10 (containing 64, 181, and 173 DAMs, respectively) were significantly enriched in the pericarp (P) and fruits (MF), primarily comprising amino acids, flavonoids, and organic acids. Clusters 3 and 8 (126 and 111 DAMs) were upregulated specifically in the roots (R), dominated by terpenoids and amino acids. Clusters 4, 5, 7, and 9 (129, 41, 181, and 115 DAMs) showed high accumulation in the seeds (S), with terpenoids, amino acids, and fatty acids as the dominant classes.

Volcano plots of six differential metabolite groups (R vs. P, R vs. MF, P vs. MF, P vs. S, S vs. R, and S vs. P) and KEGG enrichment analysis (**Supplementary Figure S2**) showed: P vs. MF had 51 upregulated and 195 downregulated DAMs, enriched in valine/leucine/isoleucine biosynthesis and flavonoid biosynthesis; S vs. R had 437 upregulated and 215 downregulated DAMs, enriched in terpenoid skeleton biosynthesis, flavonoid/flavonol synthesis, and α-linolenic acid metabolism; R vs. MF had 133 upregulated and 450 downregulated DAMs, enriched in tropane alkaloid biosynthesis, histidine metabolism, and unsaturated fatty acid synthesis.

### 3.3 Dynamic Reprogramming of the Fruit Metabolome across Ripening Stages

In fruits at distinct maturation stages, the relative abundance of soluble sugars (trehalose, sucrose, raffinose, 6-deoxy-D-glucose, and α-lactose) exhibited an increasing trend from the initial fruit (IF) to the coloration (CF), followed by a decrease at the mature fruit (MF) (**Figure 2A**). In contrast, xylitol, stachyose, nystose, α-lactose (lactose), maltotetraose, and maltotriitol showed decreasing trends across maturation stages, stabilizing in later phases. The relative expression levels of adenosine and guanosine decreased initially from stage IF to MF and then slightly rebounded, whereas 5′-S-methyl-5′-thioadenosine displayed an initial increase followed by a decrease. L-lysine, D-(+)-pipecolic acid, ornithine, and L-(+)-citrulline exhibited a rise-and-fall pattern during fruit development.

**Figure 3:**
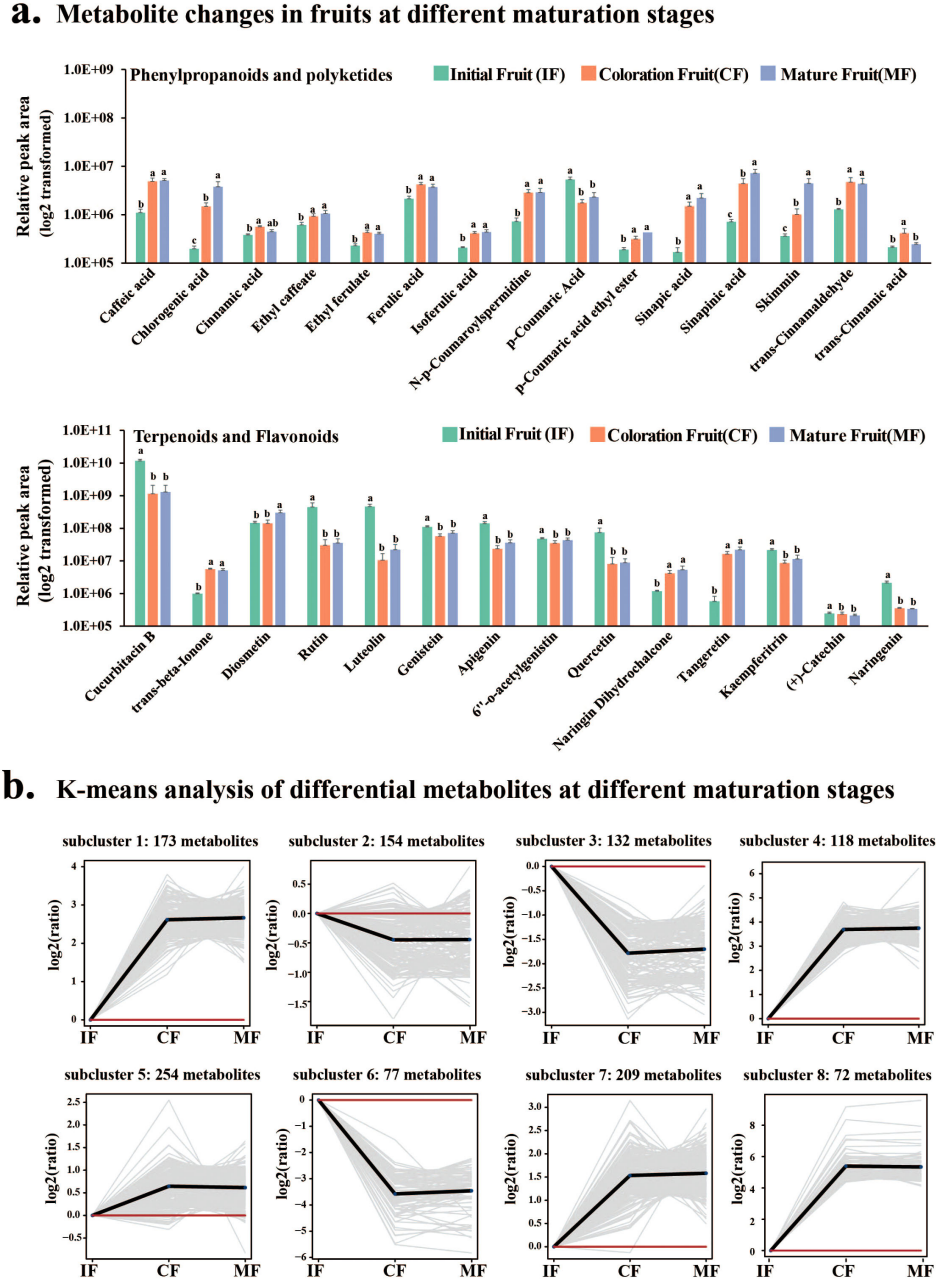
Dynamic changes of metabolites during fruit ripening. **(A)** Accumulation of phenylpropanoid, flavonoid, and terpenoid metabolites at IF, CF, and MF stages. Y-axis: Relative peak area. Different lowercase letters indicate significant differences at *p*<0.05 (Duncan’s multiple comparison; n=6 biological replicates). Cucurbitacin B decreases by 88.9% from IF to MF, while *β*-ionone increases by 4.2-fold (*p*<0.05). **(B)** K-means clustering of DAMs during ripening, divided into 8 clusters. Clusters 1/4/5/7/8 (peak at CF), Clusters 2/3/6 (decline at CF).

K-means clustering partitioned fruit metabolites into 8 clusters (Cluster 1–8) (**Figure 3B**), with dynamic patterns categorized into two groups: (1) accumulation peaking at the coloration stage (CF) followed by stabilization (Clusters 1, 4, 5, 7, and 8); (2) a decline at stage CF with sustained low levels (Clusters 2, 3, and 6). DAMs predominantly included flavonoids, terpenoids, and amino acids. IF was characterized by enrichment of primary metabolites (amino acids and sugars); CF featured significant accumulation of secondary metabolites (phenylpropanoids: sibiricose A5; phenolic acids: sinapic acid, chlorogenic acid) (**Figure 3A**); MF showed a surge in terpenoids (*β*-ionone) and alkaloids.

Flavonoids displayed dynamic accumulation patterns: kaempferitrin, quercetin, (+)-catechin, apigenin, naringenin, luteolin, and rutin decreased from stage IF to CF (fold change=0.02–0.95, *p* < 0.05) but slightly rebounded at stage MF, whereas diosmetin, tangeretin, and naringin dihydrochalcone continuously accumulated (fold change=1.0–2.1, *p* < 0.05; **Figure 3B**). Cucurbitacin B content decreased by 88.9% from stage IF to MF, while *β*-ionone increased by 4.2 folds (*p* < 0.05, n=6).

### 3.4 Transcriptomic overview and identification of the DEGs

To elucidate the gene expression patterns in different *T. kirilowii* tissues and in *T. kirilowii* fruits during different ripening stages and to identify key genes involved in the synthesis of its core bioactive components, 18 cDNA libraries were constructed (3 biological replicates per sample), yielding 43.15 million high-quality clean reads with a total data volume of 129.46 GB. Quality assessment showed Q20 values ranging from 98.42% to 99.00%, Q30 values from 95.93% to 96.94%, and GC content between 42.09% and 44.33% (**Supplementary Table S3**). *De novo* assembly using Trinity software generated 84,165 unigenes, among which 25,167 (29.9%) were longer than 1000 bp (**Supplementary Table S4**).

Six inter-tissue comparison groups (MF_vs_R, MF_vs_P, R_vs_P, MF_vs_S, R_vs_S, and P_vs_S) identified 36,741 DEGs. P_vs_MF exhibited the fewest DEGs (74 upregulated and 280 downregulated), while S_vs_P displayed the highest (6,802 upregulated and 4,869 downregulated) (**Supplementary Figure S2**). Three fruit maturation comparative groups (IF_vs_CF, IF_vs_MF, and CF_vs_MF) revealed 1,421 DEGs, with CF_vs_MF showing minimal differential expression (20 upregulated and 28 downregulated) (**Supplementary Figure S3**).

Gene Ontology (GO) enrichment analysis categorized DEGs into Biological processes (BPs), Cellular components (CCs), and Molecular functions (MFs) (**Supplementary Figure S4**). Kyoto Encyclopedia of Genes and Genomes (KEGG) pathway annotation classified 36,741 DEGs into six functional modules with significant enrichment in secondary metabolite pathways including phenylpropanoid biosynthesis (ko00940), starch and sucrose metabolism (ko00500), terpenoid backbone biosynthesis (ko00900), diterpenoid biosynthesis (ko00904), sesquiterpenoid and triterpenoid biosynthesis (ko00909), and flavonoid biosynthesis (ko00941).

### 3.5 Tissue-preferential gene expression profiles underpinning terpenoid diversification

**Figure 4:**
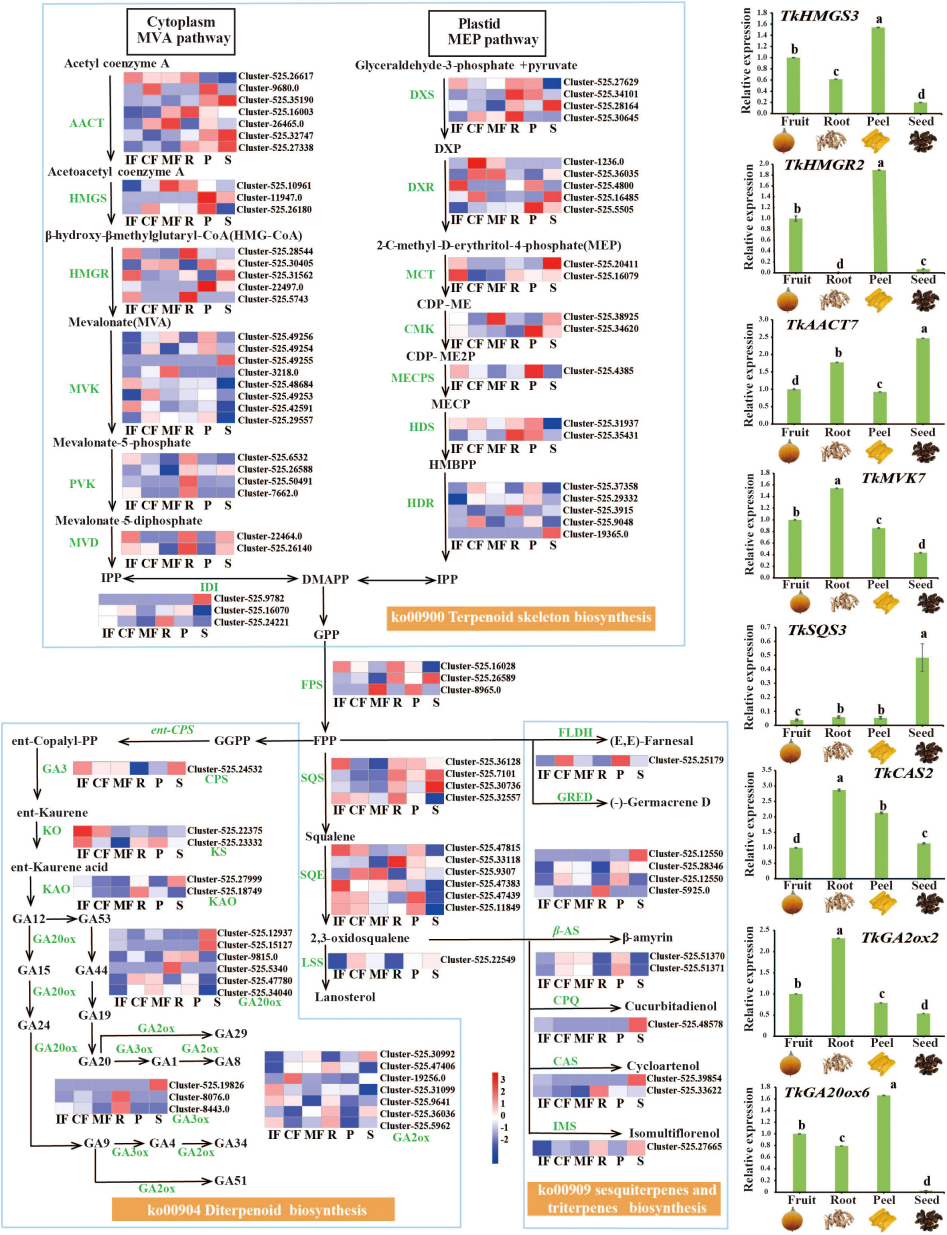
Identification of terpenoid biosynthetic genes and their expression profiles. Left: Differential terpenoid metabolic pathway map. Heatmap shows gene expression levels (FPKM) across stages (IF/CF/MF) and tissues (R/P/S). Red=high expression, blue=low expression. Right: qRT–PCR validation of 8 key terpenoid biosynthetic genes (e.g., *HMGR*, *SQS*). Reference gene: *TkGAPDH*. Relative expression levels calculated by 2^-ΔΔCT^ method. Different lowercase letters indicate significant differences at *p*<0.05 (n=3 technical replicates).

Integrated transcriptomic and metabolomic analyses identified 101 key genes involved in terpenoid biosynthesis, spanning the MVA (mevalonate) pathway, MEP (methylerythritol phosphate) pathway, 2,3-oxidosqualene pathway, and diterpene/triterpene biosynthesis pathways (**Figure 4**).

During fruit maturation, terpenoid synthesis exhibited stage-specific regulation: early MEP pathway genes (*DXS* (1-deoxy-D-xylulose-5-phosphate synthase), *DXR* (1-deoxy-D-xylulose-5-phosphate reductoisomerase), and *HDR* (4-hydroxy-3-methylbut-2-enyl diphosphate reductase); Cluster-525.30645/1236.0/9048) peaked at stage CF. MVA pathway core genes (*AACT* (acetyl-CoA acetyltransferase), *HMGS* (3-hydroxy-3-methylglutaryl-CoA synthase), *MVK* (mevalonate kinase); Cluster-26465.0/525.10961/3218.0) and downstream genes (*FPS* (farnesyl diphosphate synthase); Cluster-8965.046) were significantly upregulated at stage MF, consistent with increased terpenoid diversity in MF. This shift from MEP- to MVA-dominated biosynthesis was accompanied by distinct changes in fruit terpenoid profiles, consistent with observations in other plant species where MVA/MEP pathway partitioning regulates terpenoid diversity (Vranová et al., 2013).

Tissue-specific analyses revealed seed-specific upregulation of MVA pathway genes (*AACT*, *FPS*; Cluster-525.35190/26589) and triterpene synthesis genes (*SQS* (squalene synthase), *CPQ* (cycloartenol synthase), *CAS* (cucurbitadienol synthase), *IMS* (isomultiflorenol synthase); Cluster-525.30736/48578/39854/27665), along with elevated expression of early MEP pathway genes (*DXS* (1-deoxy-D-xylulose-5-phosphate synthase), *DXR* (1-deoxy-D-xylulose-5-phosphate reductoisomerase), *MCT* (2-C-methyl-D-erythritol 4-phosphate cytidylyltransferase); Cluster-525.28164/16485/20411/27665). In roots, high expression of MVA pathway genes (*AACT*, *HMGR* (3-hydroxy-3-methylglutaryl-CoA reductase), *PVK* (phosphomevalonate kinase), *MVD* (mevalonate diphosphate decarboxylase); Cluster-525.16003/5743/50491/26140), MEP pathway genes (*DXS*, *HDS* (4-hydroxy-3-methylbut-2-enyl diphosphate synthase), *HDR* (4-hydroxy-3-methylbut-2-enyl diphosphate reductase); Cluster-525.30645/35431/3915), and downstream terpenoid synthesis genes (*FPS*, *SQS*, and *SQE* (squalene epoxidase); Cluster-525.16028/32557/33118) was observed. In the pericarps, MEP pathway genes (*DXR*, *CMK* (4-diphosphocytidyl-2-*C*-methyl-*D*-erythritol kinase), *MECPS* (2-*C*-methyl-*D*-erythritol 2,4-cyclodiphosphate synthase); Cluster-525.5505/34620/4385) and monoterpene synthase *FLDH* [(*E, E*)-farnesal dehydrogenase, Cluster-525.25179] were activated. qRT–PCR validation confirmed agreement of the candidate gene expression (e.g., *HMGR* and *SQS*) and RNA-Seq data.

### 3.6 Stage- and tissue-specific regulation of phenylpropanoid biosynthesis genes

**Figure 5:**
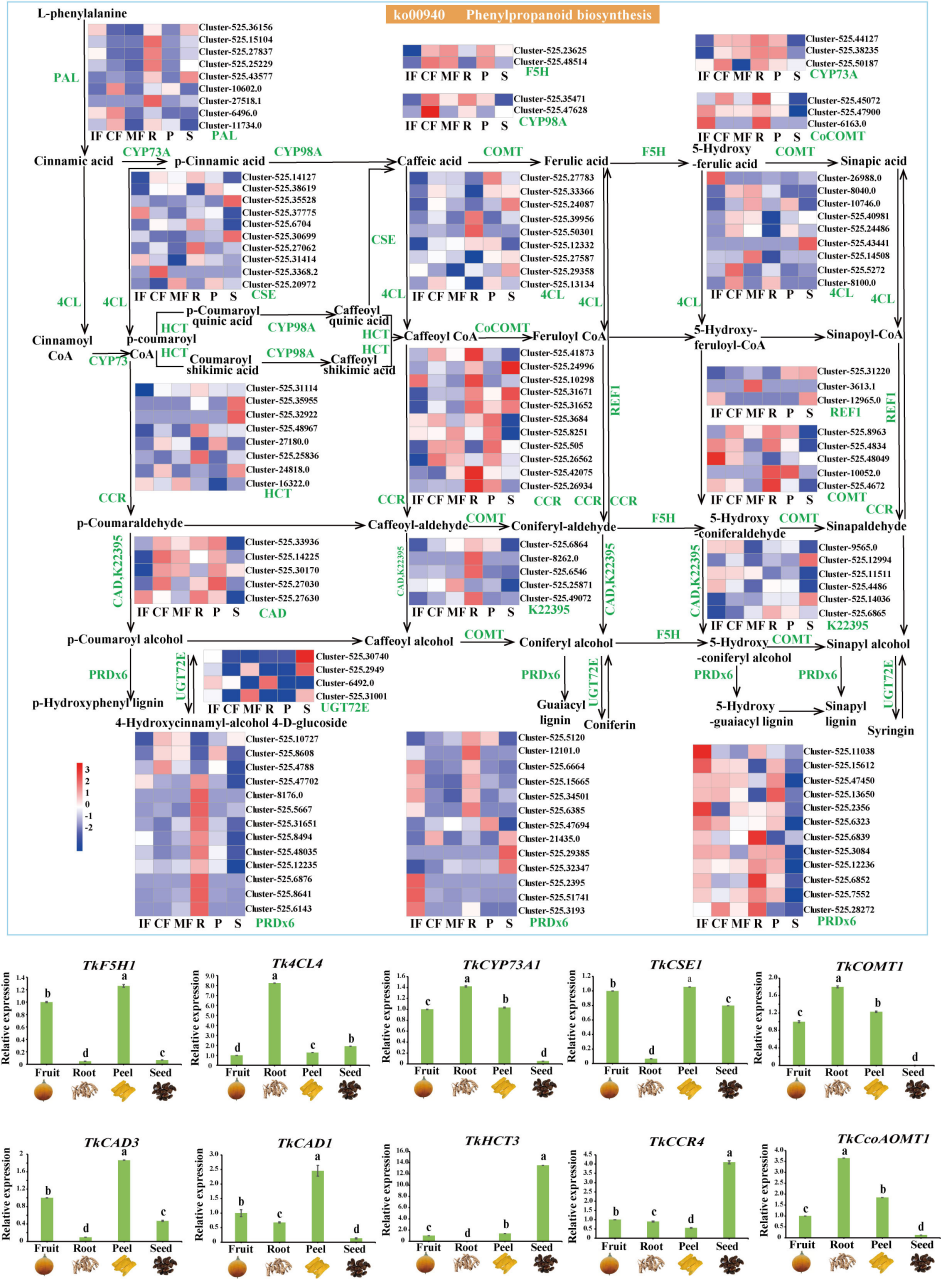
Phenylpropanoid biosynthetic pathway genes and their expression validation. UP: Differential phenylpropanoid pathway map with heatmap of 133 key genes (e.g., PAL, 4CL, COMT) across samples. Down: qRT–PCR validation of 10 candidate genes. Reference gene: *TkGAPDH*. Data are presented as mean ± SD (n=3). Different lowercase letters indicate significant differences at *p*<0.05.

Integrated transcriptomic and metabolomic analyses revealed 133 key phenylpropanoid biosynthesis pathway genes in *T. kirilowii*, encompassing 16 core enzymes (e.g., *PAL* (phenylalanine ammonia-lyase), *4CL* (4-coumarate-CoA ligase), and *COMT* (caffeic acid O-methyltransferase) in the phenylpropanoid biosynthesis pathway (ko00940) (**Figure 5**).

During fruit development, gene expression exhibited stage-specific regulation: upstream genes (*PAL* and *4CL*; Cluster-525.36156/26988) were highly expressed at stage IF; lignin biosynthesis genes (*CYP98A* (p-coumarate 3-hydroxylase) and *CAD*; Cluster-525.47628/14255) were significantly upregulated at stage CF; phenolic acid modification genes (*F5H* (ferulate 5-hydroxylase) and *COMT*; Cluster-525.48514/41873) were activated at MF, consistent with the enrichment trends of these phenolic acids in the metabolomic profiles (**Figure 3A**).

Tissue-specific analyses revealed high expression of lignin biosynthesis genes (*CCR* (cinnamoyl-CoA reductase) and *CAD* (cinnamyl alcohol dehydrogenase); Cluster-525.41873/27630) and phenolic acid-related *CYP98A* in roots (R). In mature fruits (MF), *COMT* (Cluster-525.41873) was markedly upregulated. The seeds (S) exhibited elevated *HCT* (hydroxycinnamoyl-CoA: shikimate hydroxycinnamoyl transferase, Cluster-525.32922) expression. qRT–PCR validation confirmed strong concordance between expression patterns of 10 candidate genes (e.g., *PAL*, *CYP73A* (cinnamate 4-hydroxylase), and *F5H*) and the RNA-Seq data.

### 3.7 Dynamic transcriptional regulation of flavonoid biosynthesis

**Figure 6:**
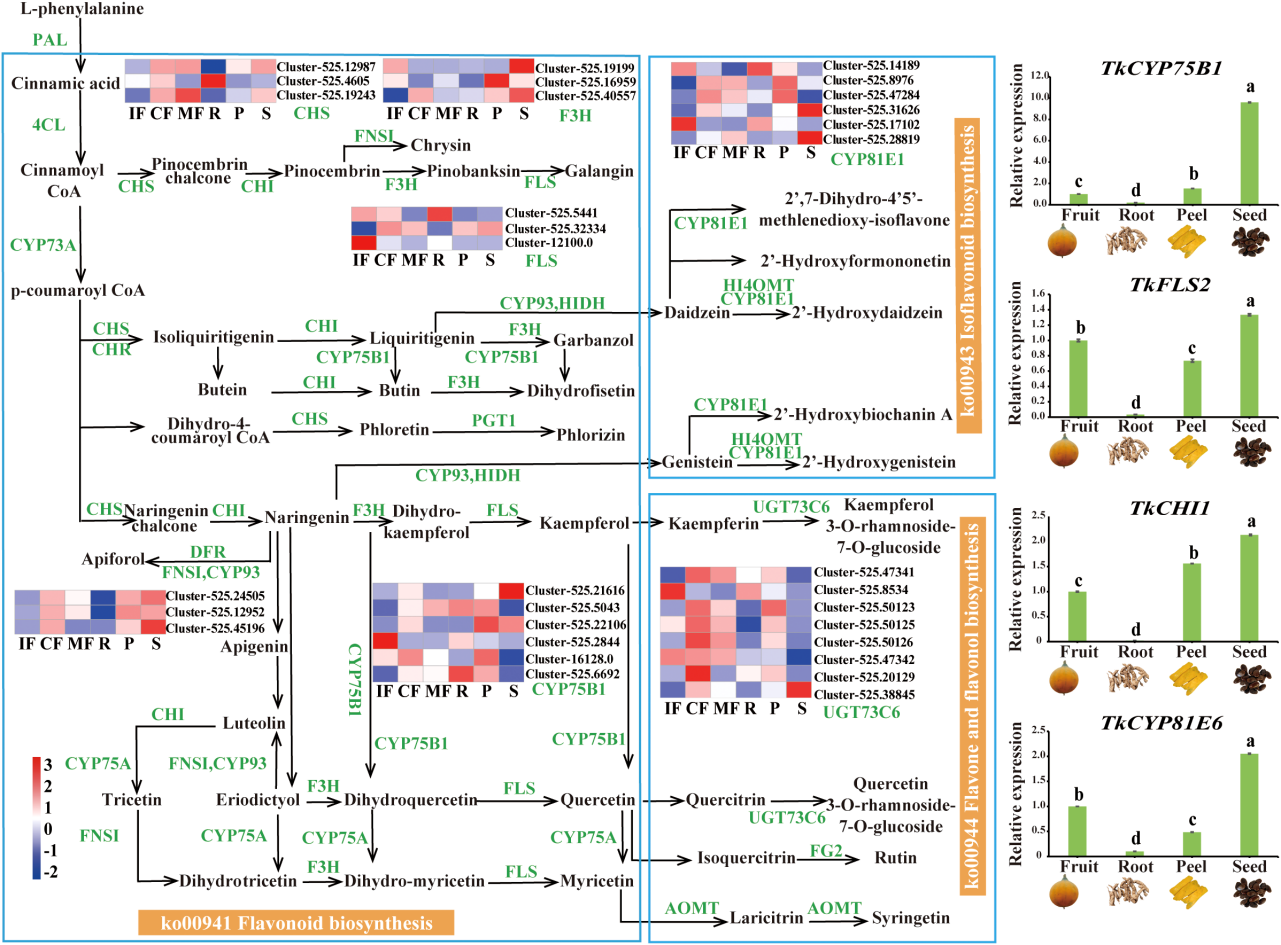
Flavonoid biosynthetic genes and their expression patterns. Left: Differential flavonoid pathway map with heatmap of 32 key genes (e.g., *CHS*, *F3H*, *UGT73C6*) across stages and tissues. Right: qRT–PCR validation of 4 candidate genes. Reference gene: *TkGAPDH*. Relative expression levels are mean ± SD (n=3). Different lowercase letters indicate significant differences at *p*<0.05.

Transcriptomic analysis identified 32 key flavonoid biosynthesis pathway genes, spanning the flavonoid (ko00941) and flavone/flavonol biosynthesis (ko00944) pathways (**Figure 6**). Flavonoid synthesis initiates from the phenylpropanoid pathway, where the upstream genes *PAL* (phenylalanine ammonia-lyase), *4CL* (4-coumarate-CoA ligase), and *CHS* (Cluster-525.12987) are highly expressed, driving the production of cinnamic acid derivatives (e.g., p-coumaroyl-CoA) to provide precursors for flavonoid core skeletons (e.g., naringenin). At stage IF, modification genes *F3H* (Cluster-525.19199), *FLS* (flavonol synthase) (Cluster-525.12100), and *CYP75B1* (Cluster-525.2844) were significantly upregulated. At stage MF, glycosyltransferase-encoding gene *UGT73C6* (UDP-glycosyltransferase 73C6) (Cluster-525.47341/8534) displayed high expression.

Tissue-specific analyses revealed elevated expression of *CHI* (chalcone isomerase) (Cluster-525.45169), *F3H* (Cluster-525.40557/19199), and *UGT73C6* (Cluster-525.38845) in the seeds (S). In the roots (R), *FLS* (Cluster-525.5441/12100.0) and *CYP75B1* (Cluster-525.2844) were specifically upregulated. qRT–PCR validation of four candidate genes (e.g., *CHS* (chalcone synthase) and *F3H*) demonstrated strong consistency with the RNA-Seq data.

### 3.8 Synergistic crosstalk between phenylpropanoid and flavonoid biosynthetic pathways

Flavonoid biosynthesis exhibited tight synergy with phenylpropanoid metabolism: cinnamic acid derivatives (e.g., p-coumaroyl-CoA) from the phenylpropanoid pathway serve as direct precursors for flavonoid backbone synthesis. Stage-specific high expression of upstream genes (*PAL*, *4CL*) at stage IF provided precursor supply. Differentiation in phenylpropanoid downstream branches (lignin/phenolic acid biosynthesis) and flavonoid pathway regulation (CF/MF) collectively shaped secondary metabolic diversity. *COMT*-mediated sinapic acid accumulation and *UGT73C6*-mediated glycosylation showed coordinated expression patterns. Phenylpropanoid-driven lignin biosynthesis and isoflavone accumulation in R, alongside flavonoid glycoside synthesis in S, demonstrated organ-specific metabolic modularity.

### 3.9 Coexpression network analysis identifies *Tk_ERF4* as a putative regulator

**Figure 7:**
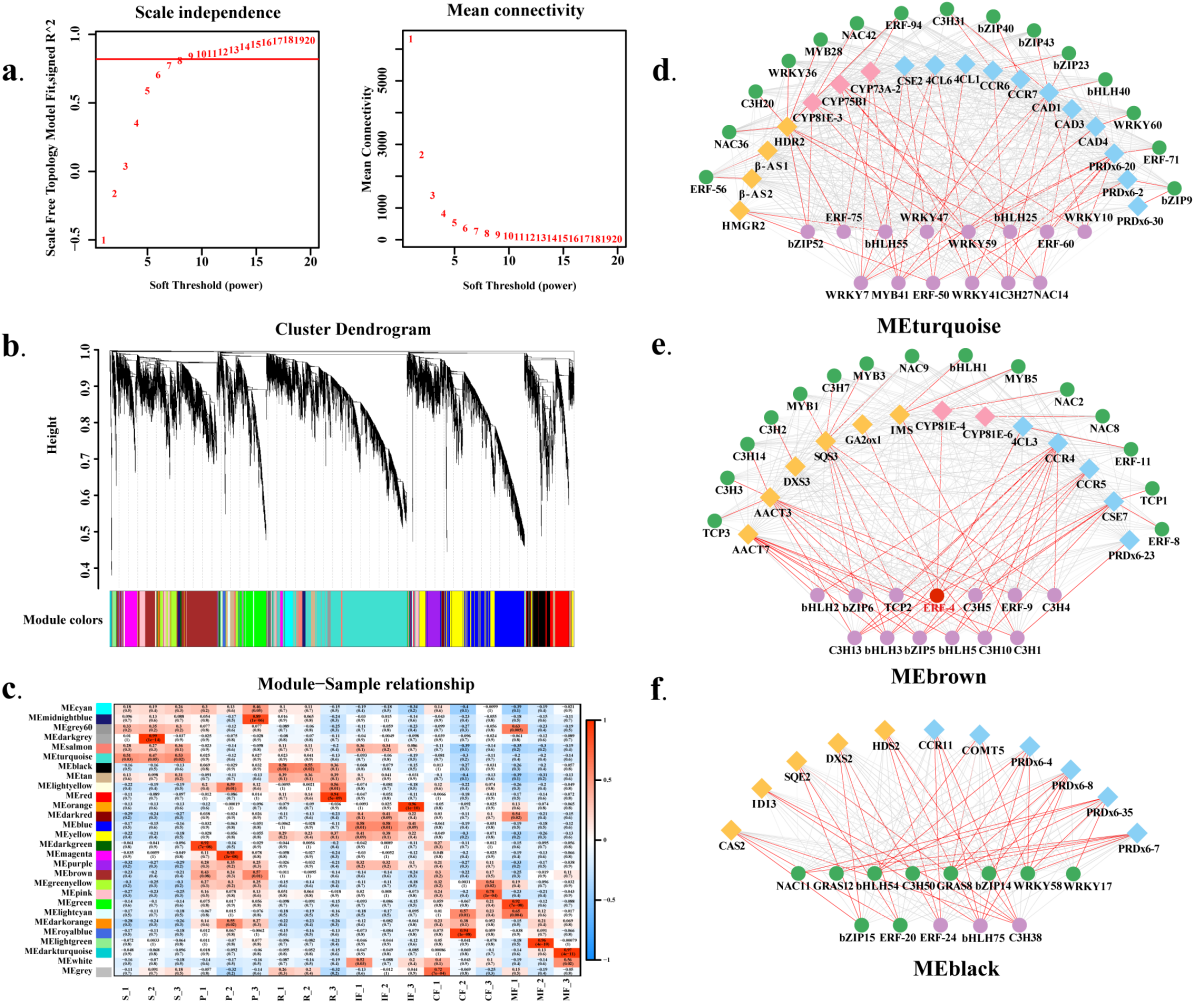
Weighted gene coexpression network analysis (WGCNA) of secondary metabolic genes. **(A)** Soft threshold selection. **(B)** Dynamic cutting tree partitioning. **(C)** Module–trait correlation analysis. **(D–F)** Visualization of transcription factor (TF) regulatory networks. Edge color intensity corresponds to correlation strength (0.8 ≤ PCCs < 1). Green/purple/red edges: TFs regulating 1/2/3 pathways (terpenoid/yellow, flavonoid/pink, phenylpropanoid/blue). Detailed gene IDs and connections are provided in **Supplementary Table 6**.

To elucidate the coexpression regulatory network underlying secondary metabolism in *T. kirilowii*, we performed weighted gene coexpression network analysis (WGCNA) using 45,000 DEGs retained after low-expression genes were filtered from the transcriptomic data. Through soft threshold screening (optimal power *β* = 8; **Figure 7A**), a dynamic cutting tree algorithm partitioned the network into 28 coexpression modules (**Figure 7B**). MEturquoise (5,082 genes) and MEblue (2,891 genes) were the largest modules, whereas MEgrey (12 genes) was the smallest.

Module-trait correlation analysis revealed that 101 terpenoid biosynthesis-related genes were significantly distributed across 13 modules, with MEturquoise (10 genes), MEblack (8 genes), and MEbrown (8 genes) showing the greatest enrichment (**Figure 7C**). Specifically, 54 terpenoid pathway genes were mapped to these 13 modules, with MEturquoise, MEblack, and MEbrown harboring the most terpenoid-related genes (10, 8, and 8, respectively). Notably, the terpenoid pathway genes enriched in these three modules (MEturquoise, MEblack, MEbrown) show clear correspondence to the tissue-specific accumulation of terpenoids in metabolomic profiles—further supporting the modular transcriptional regulation of terpenoid biosynthesis. Sixty-nine phenylpropanoid biosynthesis genes were distributed across 12 modules, with MEbrown (13 genes), MEturquoise (11 genes), and MEyellow (10 genes) showing the highest enrichment. Flavonoid metabolism-related genes (32 total) were predominantly enriched in MEbrown (9 genes) and MEdarkgrey (7 genes), further supporting the modular transcriptional regulation of secondary metabolism.

Further analysis of module-specific gene distribution analysis revealed that MEturquoise was enriched in genes related to terpenoid precursor synthesis (*AACT3*, *AACT6*, *AACT7*, and *DXS3*) and phenylpropanoid activation (*4CL3*, *4CL8*, *CYP98A-1*), indicating potential cross-pathway coordination in precursor supply. MEblack specifically harbored MVA pathway genes (*HMGR1*, *MVD2*, and *SQE2*) and phenylpropanoid initial-step genes (*CYP73A*, *4CL1*), suggesting a role in cytosolic secondary metabolite synthesis. MEbrown contained genes related to terpenoid oxidation (*β-AS1* and *GRED2*) and flavonoid modification genes (*F3H2* and *CYP81E-3*), reflecting its function in metabolite structural diversification.

Transcription factors (TFs) critically regulate the transcription of genes associated with the biosynthesis of herbal bioactive compounds by binding to their promoters, thus influencing metabolite accumulation (Huang et al., 2023; Javed and Gao, 2023). Transcription factors (TFs) from families including bHLH, AP2/ERF, bZIP, MYB, and WRKY were widely distributed in terpenoid-, phenylpropanoid-, and flavonoid-related modules. Network visualization (edge weight: 0.8 ≤ PCC < 1, FDR < 0.01) revealed key coexpression patterns:

MEturquoise: The AP2/ERF family TF *Tk_ERF4* (Cluster-525.32396) showed significant coexpression with terpenoid pathway gene *GA2ox1* (PCC=0.85), phenylpropanoid pathway gene *4CL3* (PCC=0.81), and flavonoid pathway gene *CYP81E-4* (PCC=0.84), with consistent spatiotemporal expression patterns across tissues and fruit stages (**Figure 7D**);

MEbrown: TFs including bZIP52, ERF75, and WRKY7 showed strong coexpression (PCC=0.80-0.93) with genes from two secondary metabolic pathways, indicating dual-pathway regulatory potential (**Figure 7E**);

MEblack: *Tk_ERF24* strongly interacted with terpenoid genes DXS2 (PCC=0.87) and HDS2 (PCC=0.82) and phenylpropanoid gene CCR11 (PCC=0.87), suggesting integrative regulation of terpenoid-phenylpropanoid metabolism (**Figure 7F**).

## 4 Discussion

### 4.1 Biological significance of tissue-specific metabolite accumulation

The heterogeneous distribution of metabolites across different tissues of *T. kirilowii* is significantly associated with their distinct applications and validates the traditional practice of using different plant parts for different purposes. The significant enrichment of amino acids, flavonoids, and organic acids in the fruits and pericarp provides molecular justification for their traditional use in heat-clearing and phlegm-resolving remedies, while also contributing to the fruit flavor profile and antioxidant capacity-key determinants of horticultural quality. These metabolites, particularly flavonoids like diosmetin and rutin, are well-documented for their free radical-scavenging and anti-inflammatory activities (Yu et al., 2018), which align with the fruit and pericarp’s dual role in medicine and functional foods.

Most notably, our metabolomic data unequivocally identify the root as the primary site for the biosynthesis and accumulation of cucurbitacin B (1.23×10^10^), a potent antitumor compound that inhibits STAT3 signaling pathways, induces cancer cell autophagy and apoptosis, and exhibits robust *in vitro* and *in vivo* pharmacological activities (Dai et al., 2023; Nie et al., 2024). This finding is highly consistent with the conserved tissue-specific distribution of cucurbitacin B in Cucurbitaceae crops: Hua et al. (2019) confirmed the tissue-specific synthesis of cucurbitacin B in melon, and Xu L, et al. (2022) identified a bHLH family gene Brp that specifically regulates cucurbitacin B biosynthesis in melon roots, directly supporting the root as the major synthesis site of cucurbitacin B in *T. kirilowii*. Additionally, Luo et al. (2024) clearly demonstrated that cucurbitacin B synthesis in Cucurbitaceae crops is synergistically regulated by WRKY and bHLH family transcription factors (e.g., protein interaction between CmWRKY13 and CmBt in melon), which further explains the specific enrichment of cucurbitacin B in *T. kirilowii* roots. Refined WGCNA analysis shows that the MEblack module specifically harbors MVA pathway genes (*HMGR1*, *MVD2*, *SQE2*), and cucurbitacin B is classified as a terpenoid compound, suggesting that this module may participate in root cucurbitacin B synthesis by regulating the MVA pathway—providing a potential transcriptional mechanism for this tissue-specific enrichment. This positions the root as a prioritized and sustainable source for the extraction and bioprospecting of this high-value pharmaceutical ingredient, offering a clear target for breeding programs aimed at enhancing root-specific metabolite yield. The initial-stage fruits also accumulate substantial cucurbitacin B (1.17×10^10^), suggesting they could serve as an alternative resource to reduce pressure on root harvesting, supporting the sustainable utilization of *T. kirilowii*.

Seeds exhibit the highest content of α,α-trehalose (1.2×10^8^) among all tissues. In plants, trehalose is widely recognized for its roles in stress tolerance (e.g., drought, oxidative stress) and energy storage, which likely supports seed dormancy maintenance and germination success (Wang et al., 2026; Zhang et al., 2025). Additionally, the seed’s metabolic profile—dominated by lipids (linoleic acid content >60%) and specific terpenoids—validates its dual role as a medicinally active laxative and a source of nutritional oil (Chen et al., 2024; Xu L, et al., 2022). The MEturquoise module is enriched in terpenoid precursor synthesis genes (*AACT3*, *AACT6*, *AACT7*, *DXS3*), and seeds accumulate significant levels of terpenoids, suggesting an association between this module and seed terpenoid synthesis—reflecting the characteristics of modular transcriptional regulation of seed functional metabolites. This precise metabolic partitioning provides an irrefutable scientific basis for the traditional practice of differential organ usage and delivers a set of chemical markers (e.g., cucurbitacin B for roots, diosmetin for fruits, α,α-trehalose for seeds) essential for the authentication, grading, and quality control of raw materials in commercial supply chains.

### 4.2 Dynamic reprogramming of metabolic networks during fruit ripening and implications for precision harvesting

Our multiomics data delineate a precise metabolic trajectory during fruit development, offering a science-informed guide for horticultural practices. We propose a three-phase model: an initial growth phase (IF) supported by primary metabolism (amino acids and sugars), a crucial quality-determination phase at the color-changing stage (CF) characterized by the explosive biosynthesis of phenylpropanoids (sibiricose A5, chlorogenic acid) and phenolic acids (sinapic acid), and a final ripening phase (MF) marked by the accumulation of aroma compounds (e.g., *β*-ionone) and the glycosylation of flavonoids.

Notably, metabolic divergence between CF and MF was minimal (VIP<1.0, p>0.05), with 93.4% of differentially abundant metabolites (DAMs) identified between IF and CF. This observation indicates that secondary metabolic reprogramming predominantly occurs in early fruit development—a key finding with practical implications. Transcriptomic data further support this: the IF_vs_CF comparison identified 1,373 DEGs, whereas CF_vs_MF only yielded 48 DEGs, confirming that the major transcriptional reprogramming driving secondary metabolite accumulation is completed by the color-changing stage.

WGCNA further links this early reprogramming to stage-specific module activation: MEP pathway genes in MEturquoise and MVA pathway genes in MEblack show stage-preferential expression, mirroring the conserved MVA/MEP partitioning that modulates terpenoid diversity (Vranová et al., 2013). Furthermore, this metabolic dynamics is highly consistent with the conserved developmental pattern of Cucurbitaceae crops: Hua et al. (2019) observed that cucurbitacin B in melon fruits peaks at the early developmental stage (10 DAF, similar to the IF stage of *T. kirilowii*) and then continuously decreases, which is fully consistent with the 88.9% decrease in cucurbitacin B content in *T. kirilowii* fruits from IF to MF stages; Sun et al. (2024) summarized that MYB family transcription factors in Cucurbitaceae crops regulate fruit sweetness by controlling sucrose synthesis genes during ripening, which is consistent with the accumulation mechanism of high-sweetness metabolites (e.g., naringin dihydrochalcone) in *T. kirilowii* at the MF stage. Our model suggests that harvesting at the color-changing stage is ideal for obtaining raw material with peak levels of phenolic acids and flavonoids (e.g., chlorogenic acid, quercetin), maximizing its medicinal potential. In contrast, delaying harvest until full maturity may be preferable for achieving a more desirable flavor profile, as evidenced by the metabolic shift during ripening: bitter/astringent flavonoids (naringenin, (+)-catechin) decline by 16–87%, while high-sweetness naringin dihydrochalcone accumulates 3.6–4.6 folds (**Figure 3B**). This deliberate metabolic adjustment likely improves fruit palatability and consumer acceptance of fruit-based products, a key consideration for the functional food industry.

Additionally, the significant reduction of cucurbitacin B (88.9% decrease from stage IF to MF) and accumulation of monoterpene *β*-ionone (4.2 folds increase) further contribute to bitterness mitigation and aroma enhancement, respectively (Castellaneta et al., 2025; Xu et al., 2024). These dynamic changes underscore the importance of developmental timing in determining the final quality and application potential of the fruit—either as a medicine with specific bioactive profiles or as a food with desirable sensory attributes.

### 4.3 Transcriptional regulatory networks and key targets for metabolic engineering

Moving beyond correlative observations, our WGCNA unravels the transcriptional regulatory hierarchy that governs the spatiotemporal biosynthesis of bioactive compounds. The stage-specific regulation of terpenoid synthesis—MEP pathway genes (*DXS*, *DXR*, *HDR*) peaking at stage CF and MVA pathway genes (*AACT*, *HMGS*, *MVK*) upregulated at stage MF—reflects a strategic metabolic switch. This shift from plastid-localized MEP to cytosolic MVA pathways is accompanied by distinct changes in fruit terpenoid profiles (accumulation of *β*-ionone and other terpenoids at MF), and MVA-derived terpenoids (e.g., *β*-amyrin) often serve as precursors for protective compounds and signaling molecules (Vranová et al., 2013).

Tissue-specific gene expression further reinforces metabolic specialization: seed-specific MVA pathway and triterpene synthesis genes, along with root-activated MVA/MEP pathways, align with the tissue-preferential accumulation of terpenoids—consistent with the modular transcriptional regulation revealed by WGCNA; pericarp MEP pathway genes (*DXR*, *CMK*) and monoterpene synthase FLDH facilitate volatile sesquiterpene synthesis, contributing to fruit aroma formation—a key quality trait for horticultural products.

Notably, the co-expression network identifies *Tk_ERF4* (Cluster-525.32396) as a putative master regulator, co-expressed with key genes across the terpenoid (*GA2ox1*, PCC = 0.85, FDR<0.01), phenylpropanoid (*4CL3*, PCC = 0.81, FDR<0.01), and flavonoid (*CYP81E-4*, PCC = 0.84, FDR<0.01) pathways. This multi-pathway co-expression pattern aligns with the conserved regulatory logic of secondary metabolism in Cucurbitaceae—Luo et al. (2024) directly confirmed via yeast two-hybrid (Y2H), bimolecular fluorescence complementation (BiFC) and luciferase complementation (LCI) assays that cucurbitacin B (CuB) synthesis in melon is synergistically regulated by WRKY and bHLH family TFs. Specifically, *CmWRKY13* interacts with *CmBt* at the protein level, and the complex jointly binds to the W-box/E-box elements of CuB synthesis genes (*Cm180*, *Cm160*, *Cm170*, *CmACT*) to enhance their expression. Meanwhile, CuB in melon fruits reaches peak content at 5 days after flowering (DAF, similar to the IF stage of *T. kirilowii*) and then gradually decreases, which is highly consistent with the developmental dynamic of CuB in *T. kirilowii* fruits. This verifies that “multi-family TF synergistic regulation of secondary metabolism” is a conserved mechanism in Cucurbitaceae and further supports the hypothesis that *Tk_ERF4* (AP2/ERF family) may form a regulatory complex with MYB, bHLH and other families to participate in the regulation of secondary metabolism genes in *T. kirilowii*. Furthermore, the widespread involvement of TF families such as AP2/ERF, MYB, and WRKY underscores the complexity and robustness of the regulatory network. Sun et al. (2024) systematically summarized that flavonoid synthesis in Cucurbitaceae crops follows a conserved “MYB-4CL” regulatory model—MYB transcription factors drive flavonoid accumulation in fruits by activating the key phenylpropanoid pathway gene *4CL*, which is highly consistent with the enrichment of flavonoids in *T. kirilowii* fruits and the significant co-expression between *Tk_ERF4* and *4CL3*. Meanwhile, the review also reported that *CmERF1-2* (ERF family) in melon directly represses *CmMYB44* (MYB family) to regulate sucrose accumulation and ethylene production, providing cross-species evidence for the potential interaction between *Tk_ERF4* (AP2/ERF family) and MYB family in *T. kirilowii*. WGCNA also identifies other TFs with potential pathway-specific roles: *ERF24* shows strong co-expression with terpenoid genes *DXS2* (PCC = 0.87, FDR<0.01), *HDS2* (PCC = 0.82, FDR<0.01), and phenylpropanoid gene *CCR11* (PCC = 0.87, FDR<0.01); *bZIP52* and *ERF75* exhibit strong co-expression with genes from two secondary metabolic pathways (PCC = 0.80–0.93, FDR<0.01)—suggesting a hierarchical regulatory pattern involving multi-pathway master regulators and pathway-specific factors. These TFs constitute a valuable genetic toolkit for the molecular breeding of *T. kirilowii*. Employing gene editing technologies (e.g., *CRISPR/Cas9*) in the future to fine-tune the expression of these TFs represents a promising strategy for developing new cultivars with optimized medicinal component profiles in specific tissues, ultimately increasing the value and efficacy of this horticultural crop.

### 4.4 Metabolic pathway crosstalk and practical implications

The interconnectedness of the phenylpropanoid, flavonoid, and terpenoid pathways, as revealed by our integrated analysis, highlights the synergistic nature of secondary metabolism in *T. kirilowii*. Flavonoid biosynthesis exhibits tight synergy with phenylpropanoid metabolism: cinnamic acid derivatives (e.g., p-coumaroyl-CoA) from the phenylpropanoid pathway serve as direct precursors for flavonoid backbone synthesis. Moreover, upstream genes of both pathways (*PAL*, *4CL* in phenylpropanoids; *CHS*, *F3H* in flavonoids) show consistent high expression at the IF stage, indicating coordinated transcriptional regulation of precursor supply. This shared precursor pool ensures efficient resource allocation, as plants can redirect metabolites between pathways based on developmental or environmental demands.

Differentiation of downstream branches of the phenylpropanoid pathway (e.g., lignin/phenolic acid biosynthesis) and stage-specific regulation of the flavonoid pathway (CF/MF) collectively shape secondary metabolic diversity. The findings of this study can be translated into three actionable applications: 1) Precision harvesting: the color-changing stage (CF) is the optimal harvest time for fruit-based medicinal products, as this stage has peak flavonoid and phenolic acid content; 2) Quality control: tissue-specific markers (cucurbitacin B for roots, diosmetin for fruits, α,α-trehalose for seeds) enable accurate raw material authentication; 3) Molecular breeding: *Tk_ERF4*, *ERF24*, and *bZIP52* can serve as candidate targets for subsequent functional validation, providing new directions for addressing the long-standing challenge of “single-trait improvement” in medicinal plant breeding.

Future research should focus on the effects of environmental factors (e.g., light, abiotic stress) on this regulatory network, to deepen understanding of metabolic plasticity in *T. kirilowii* and provide scientific support for developing agronomic practices that further optimize target bioactive compound yields. This pathway crosstalk, mediated by shared precursors and coordinated TF regulation, must be considered in any metabolic engineering or crop improvement strategy—the tissue-specific genes and key transcription factors identified in this study provide a prioritized list of genetic targets for marker-assisted selection or genetic engineering to tailor metabolic profiles of specific organs.

## 5 Conclusion

In conclusion, our integrated multiomics study provides a comprehensive spatiotemporal atlas of metabolism and gene regulation in *T. kirilowii*. We molecularly validated the principle of “using different parts for different purposes” through delineation of the tissue-specific accumulation of the following medicinal compounds: cucurbitacin B in roots, fatty acids and terpenoids in seeds, and flavonoids in fruits/pericarp—a pattern that correlates with the enrichment of secondary metabolism-related genes in MEblack, MEturquoise, and MEbrown modules as revealed by refined WGCNA. Furthermore, we elucidated the dynamic metabolic reprogramming that occurs during fruit ripening, revealing that the color-changing stage is a critical window for secondary metabolism, providing a scientific basis for precision harvesting. Most importantly, our coexpression network analysis revealed key transcriptional regulators, notably *Tk_ERF4* as a putative multi-pathway coordinator and *ERF24*/*bZIP52* as pathway-specific modulators, that orchestrate the synergistic biosynthesis of major bioactive compounds.

This work transcends fundamental description, providing a genetic and biochemical roadmap for the future horticultural improvement of *T. kirilowii*. The datasets and insights generated here will be indispensable for breeding high-quality varieties, establishing objective quality standards, and developing sustainable bioproduction strategies for their active ingredients. As a paradigm for multiomics research in medicinal horticulture crops, this study not only reveals the potential of *T. kirilowii* but also sets a benchmark for the scientific exploration of other medicinal plants, ultimately bridging the gap between traditional herbal medicine and modern precision agriculture.

## Data Availability

The datasets presented in this study can be found in online repositories. The RNA-seq data generated in this study have been deposited in the China National Center for Bioinformation (CNCB) under the accession number CRA025465. All other data analyzed during this study are included in the article and its **Supplementary Material**.
