# Supplementary material for: Integrated multiomics profiling elucidates the spatiotemporal metabolic dynamics and regulatory networks of the bioactive components of Trichosanthes kirilowii
Source: Front Plant Sci. 2026 Feb 17;17:1735703. doi: 10.3389/fpls.2026.1735703 (PMC12953388; doi:10.3389/fpls.2026.1735703)

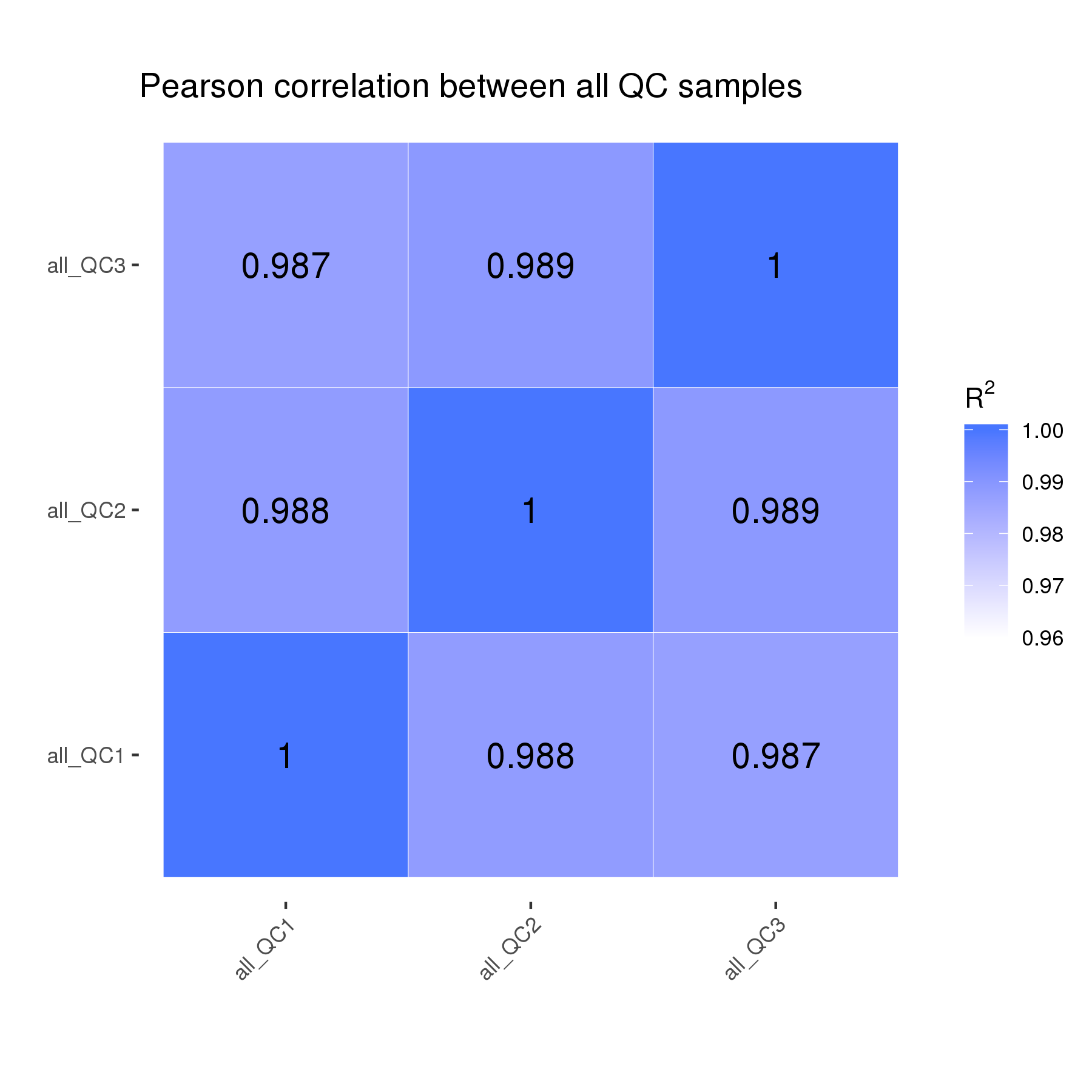
**Fig.S1** A: Heat map of (Pearson’s correlation coefficient) value between all QC samples. The color represented the p value.

**Fig.S2** Volcano plots of tissue-specific differential metabolites (group comparisons: R vs MF, R vs MF, P vs MF, R vs P, S vs R, P vs S) and KEGG enrichment analysis.

**R.vs.MF TOP 20 of KEGG Enrichiment**

**R.vs.MF Differential metabolite volcano map**


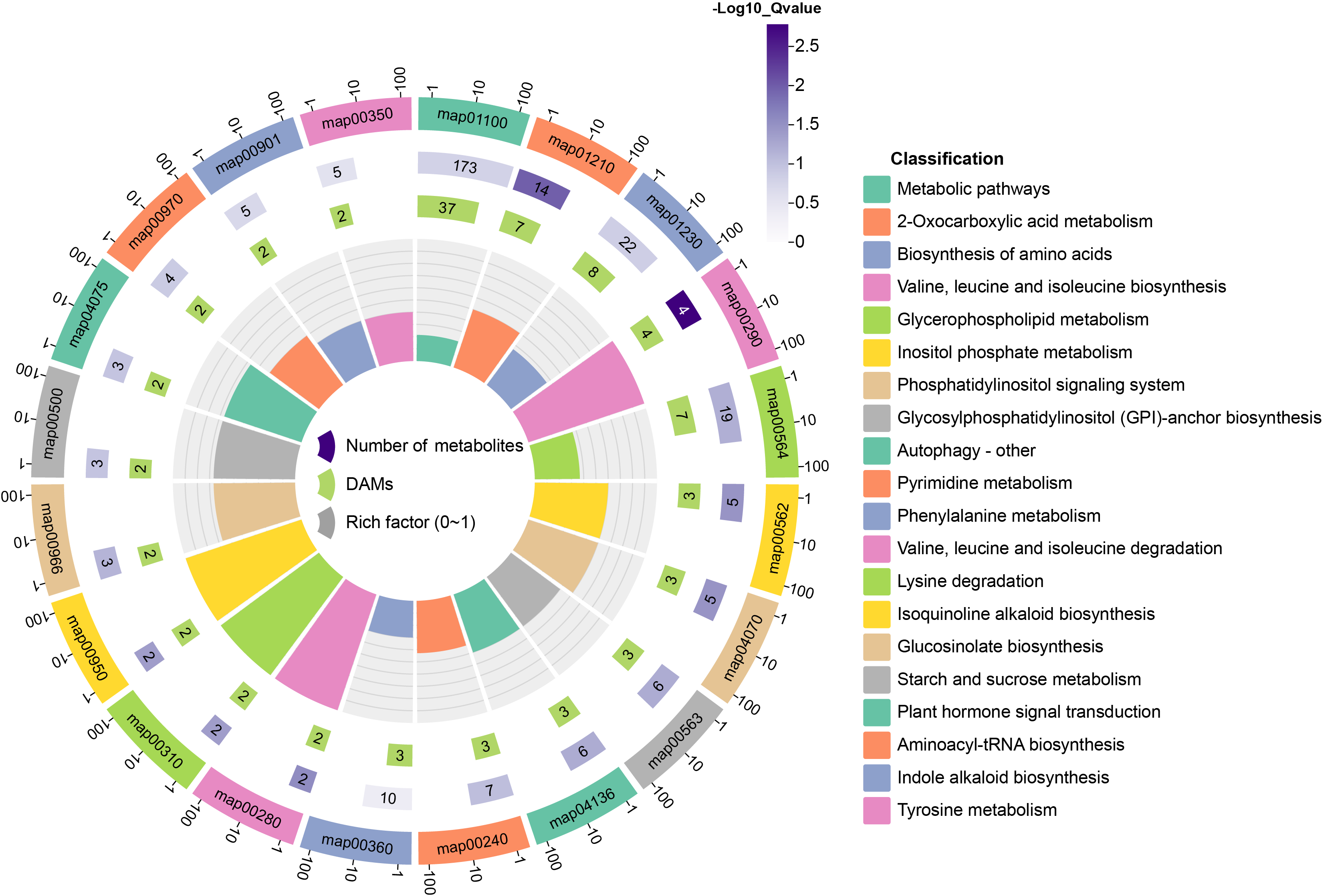

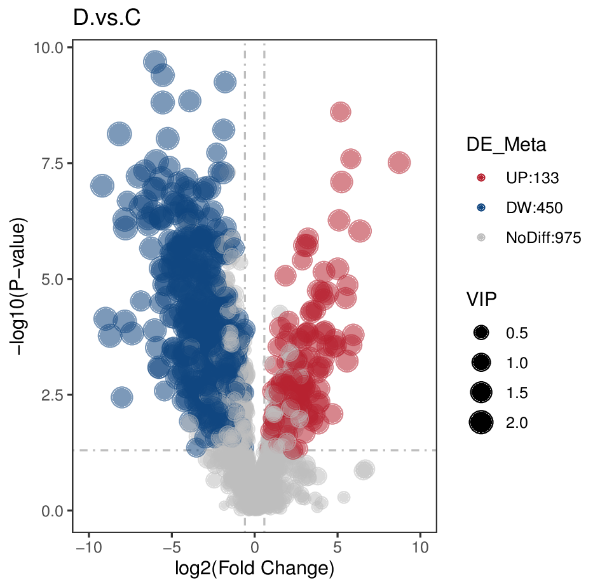


**S.vs.MF TOP 18 of KEGG Enrichiment**

**S.vs.MF Differential metabolite volcano map**


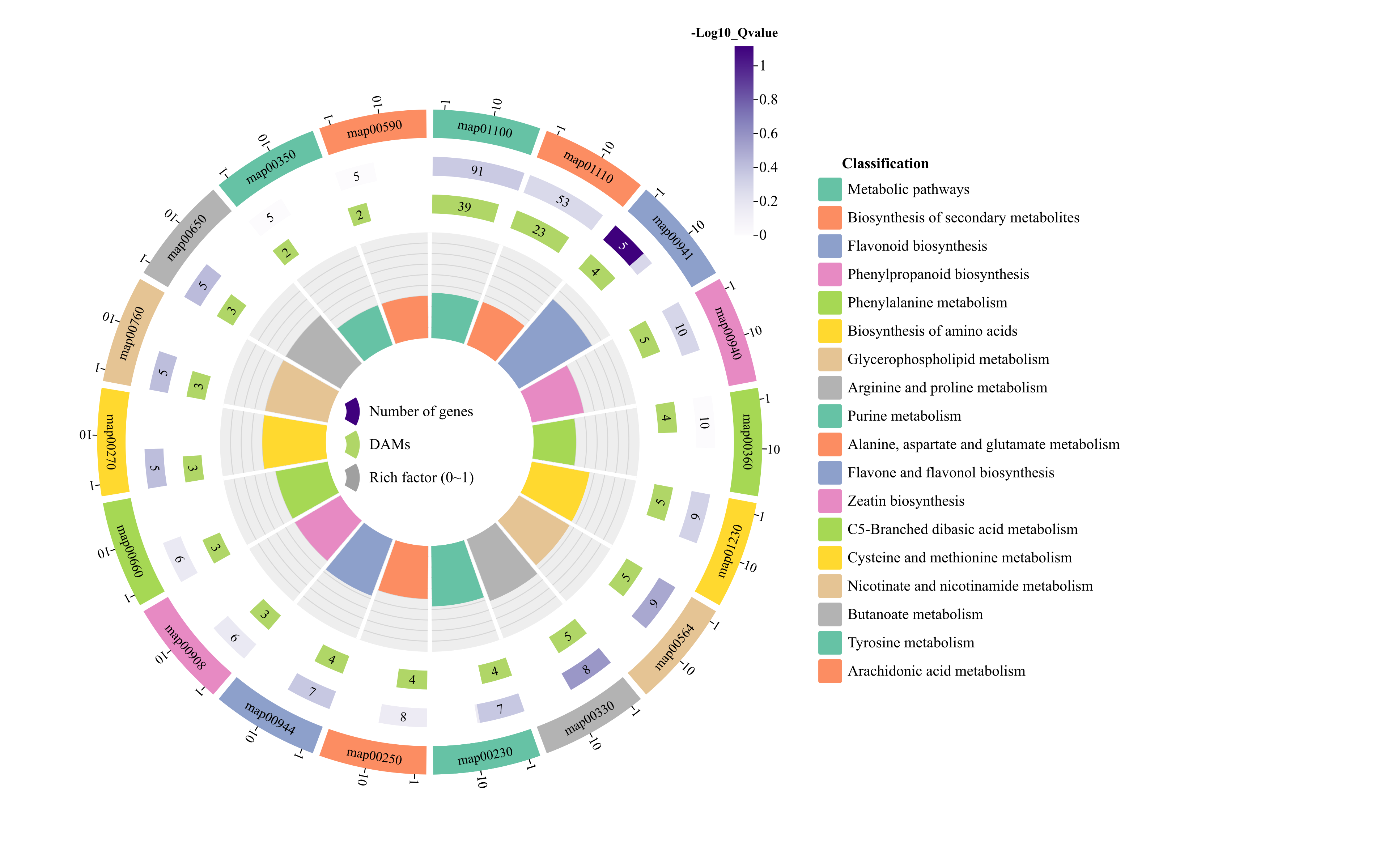

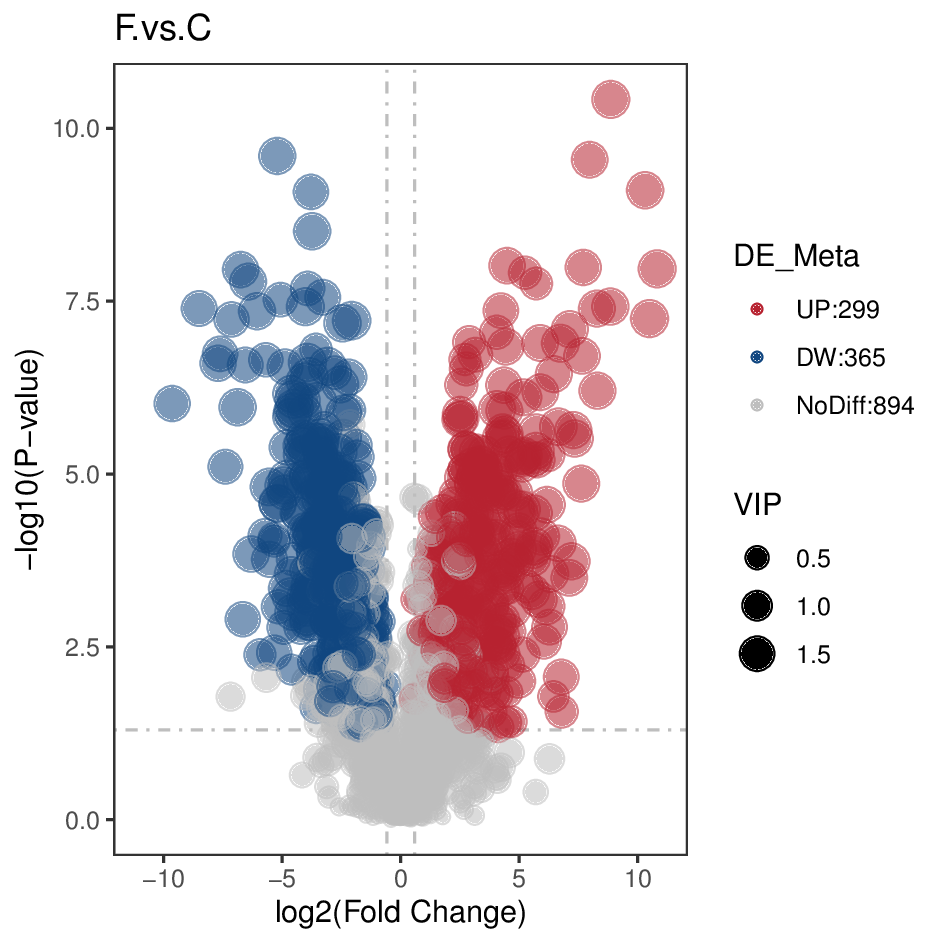


**P.vs.MF TOP 20 of KEGG Enrichiment**


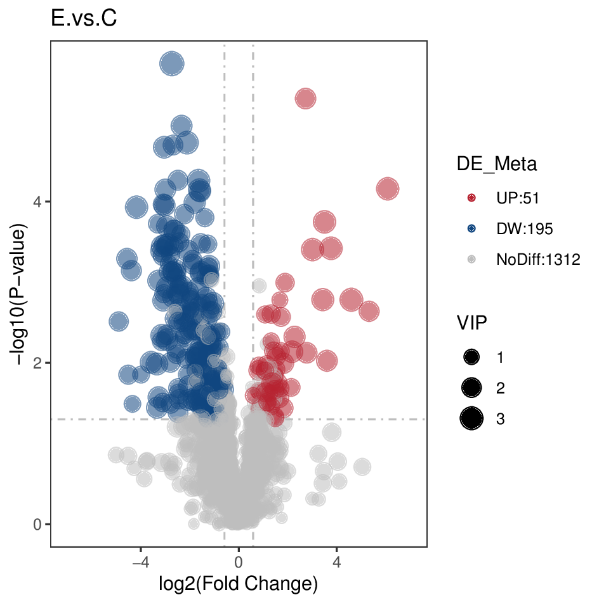


**P.vs.MF Differential metabolite volcano map**


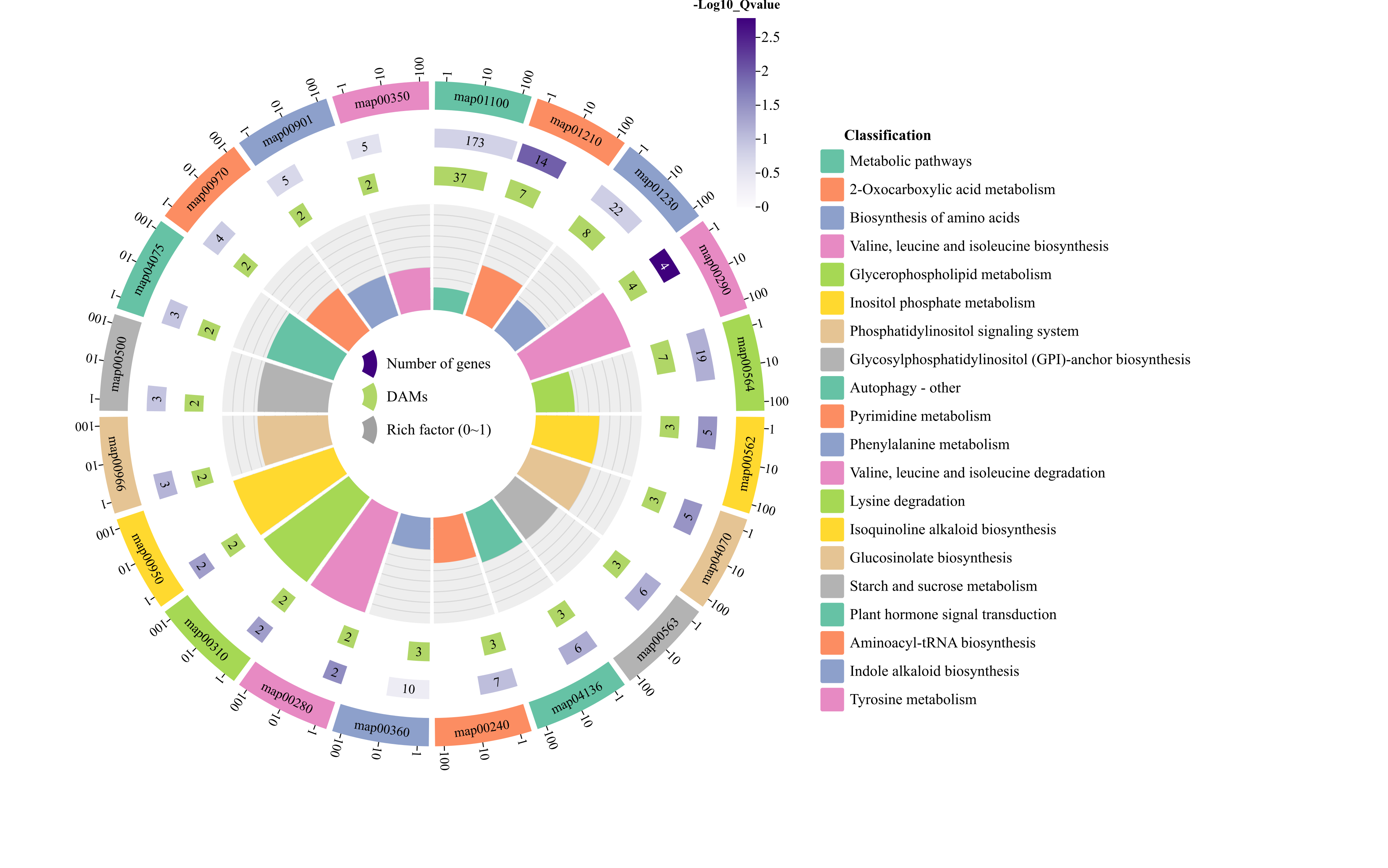


**P.vs.S TOP 20 of KEGG Enrichiment**


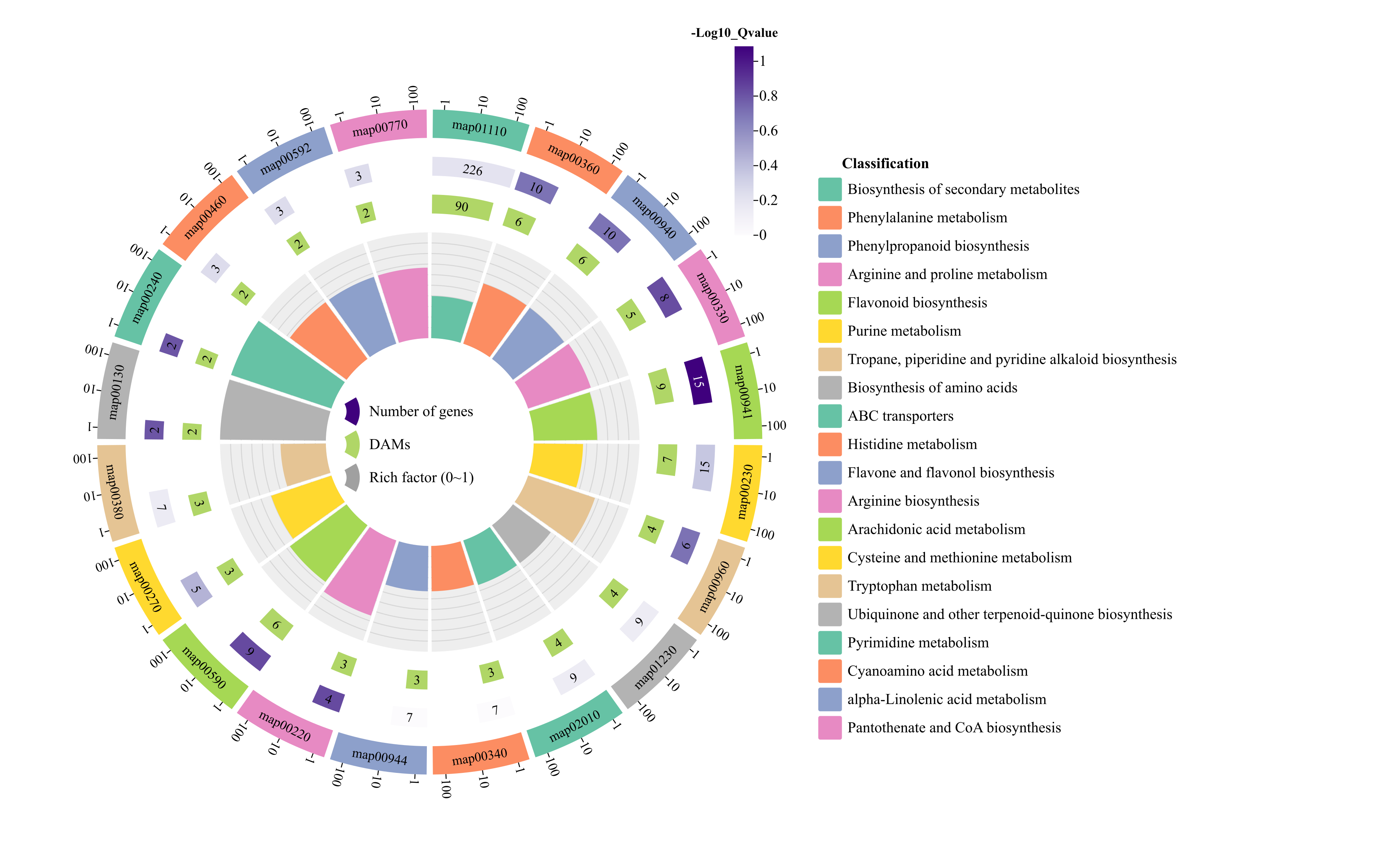

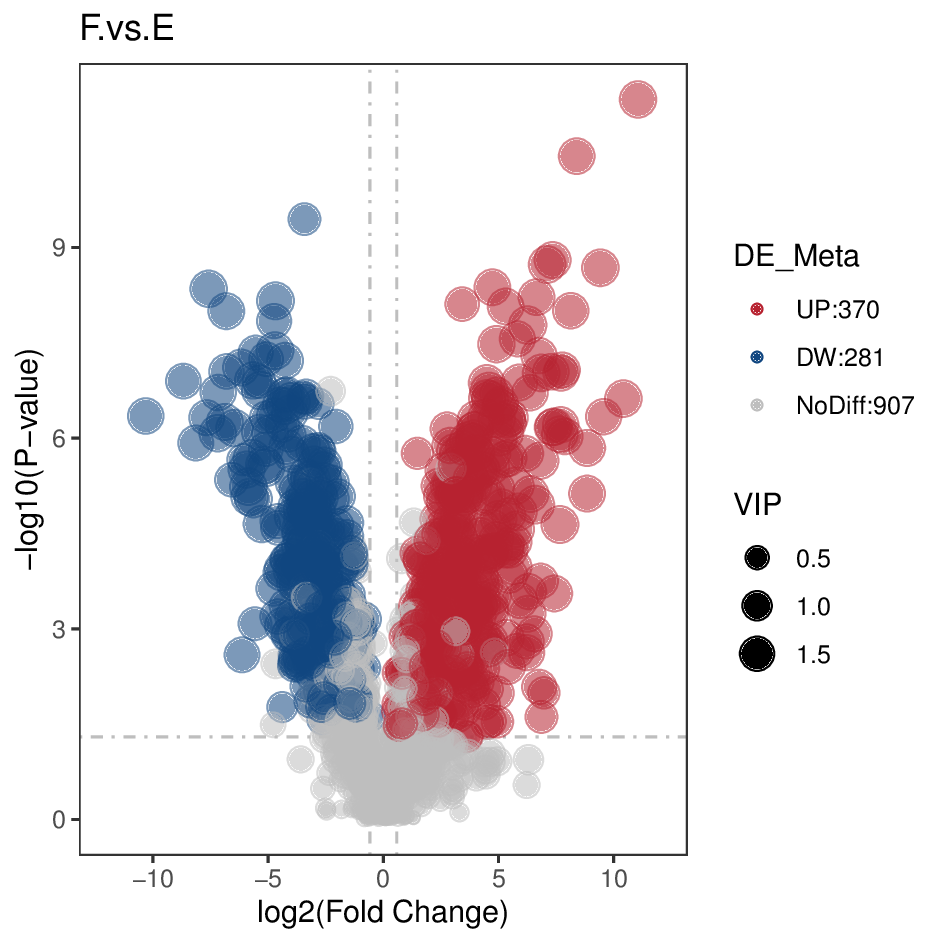


**P.vs.S Differential metabolite volcano map**

**S.vs.R TOP 20 of KEGG Enrichiment**


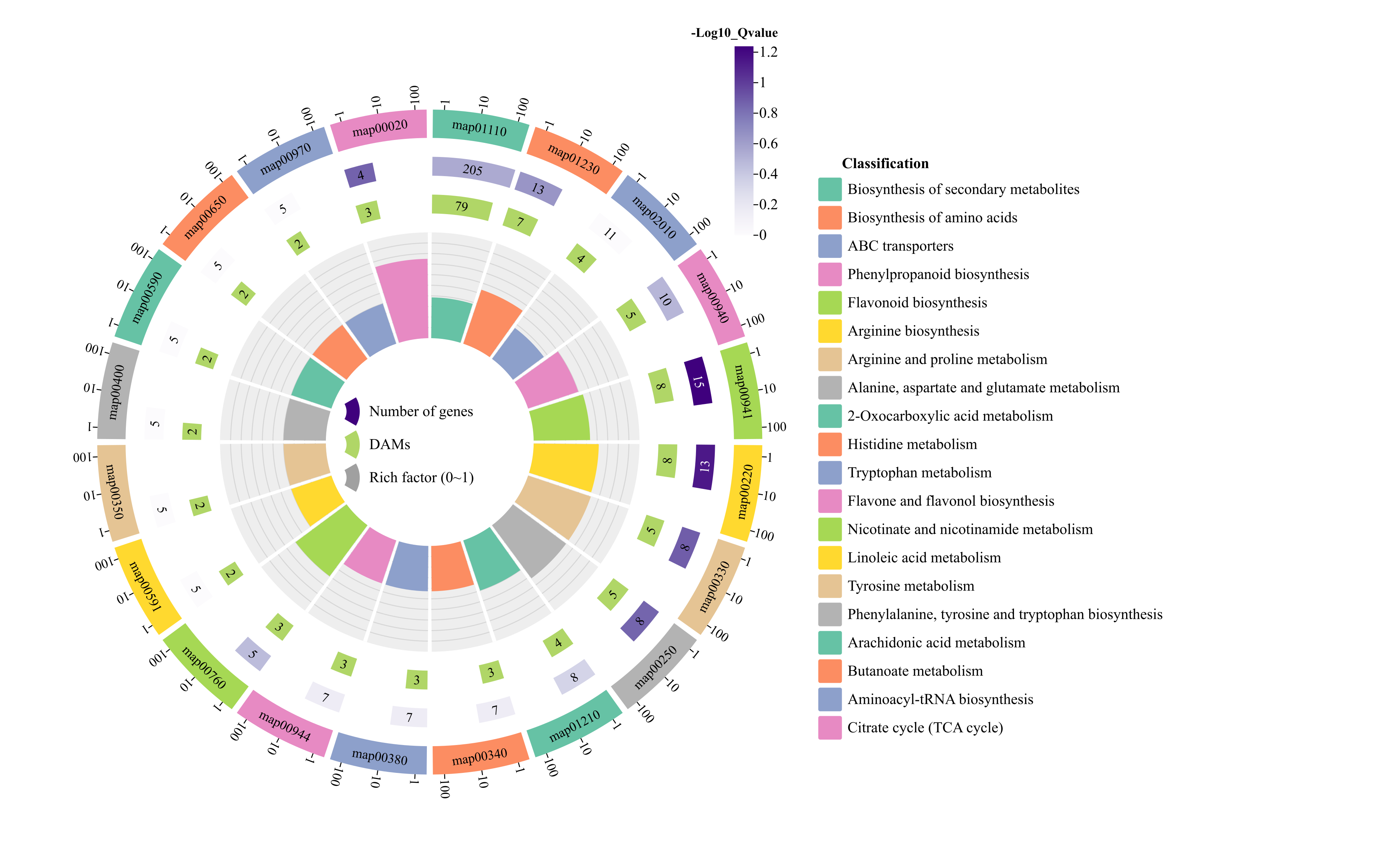

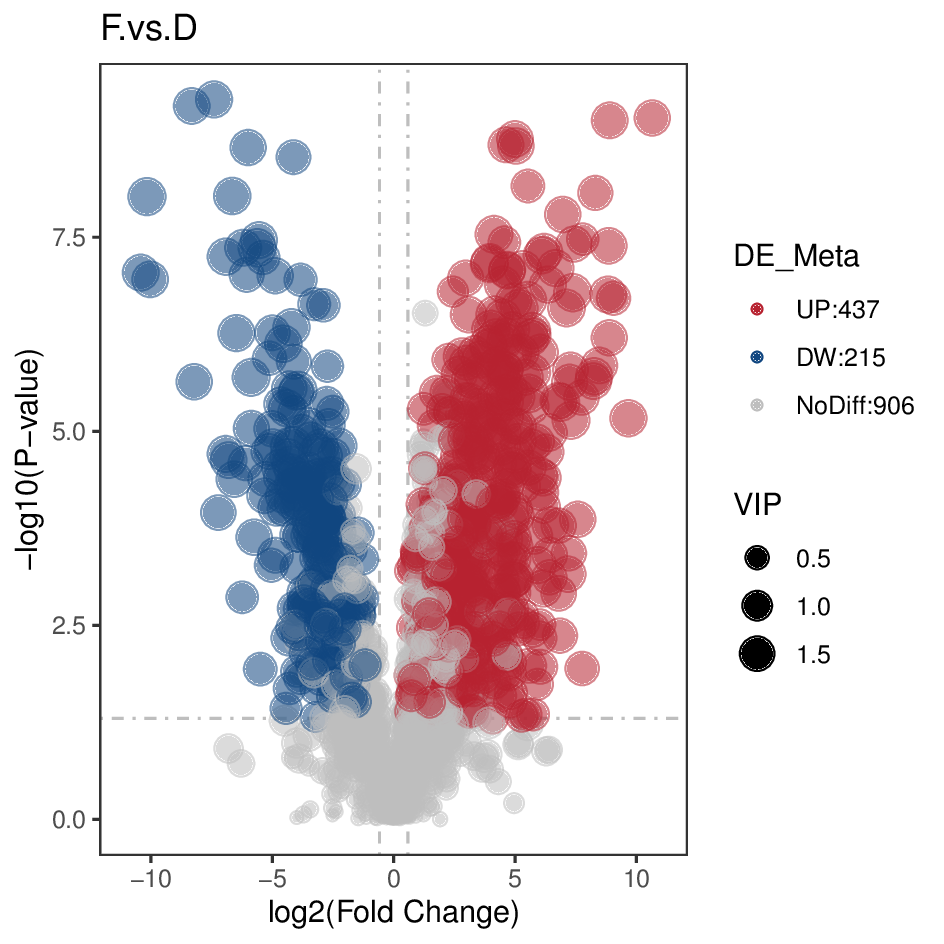


**S.vs.R Differential metabolite volcano map**

**R.vs.S TOP 20 of KEGG Enrichiment**


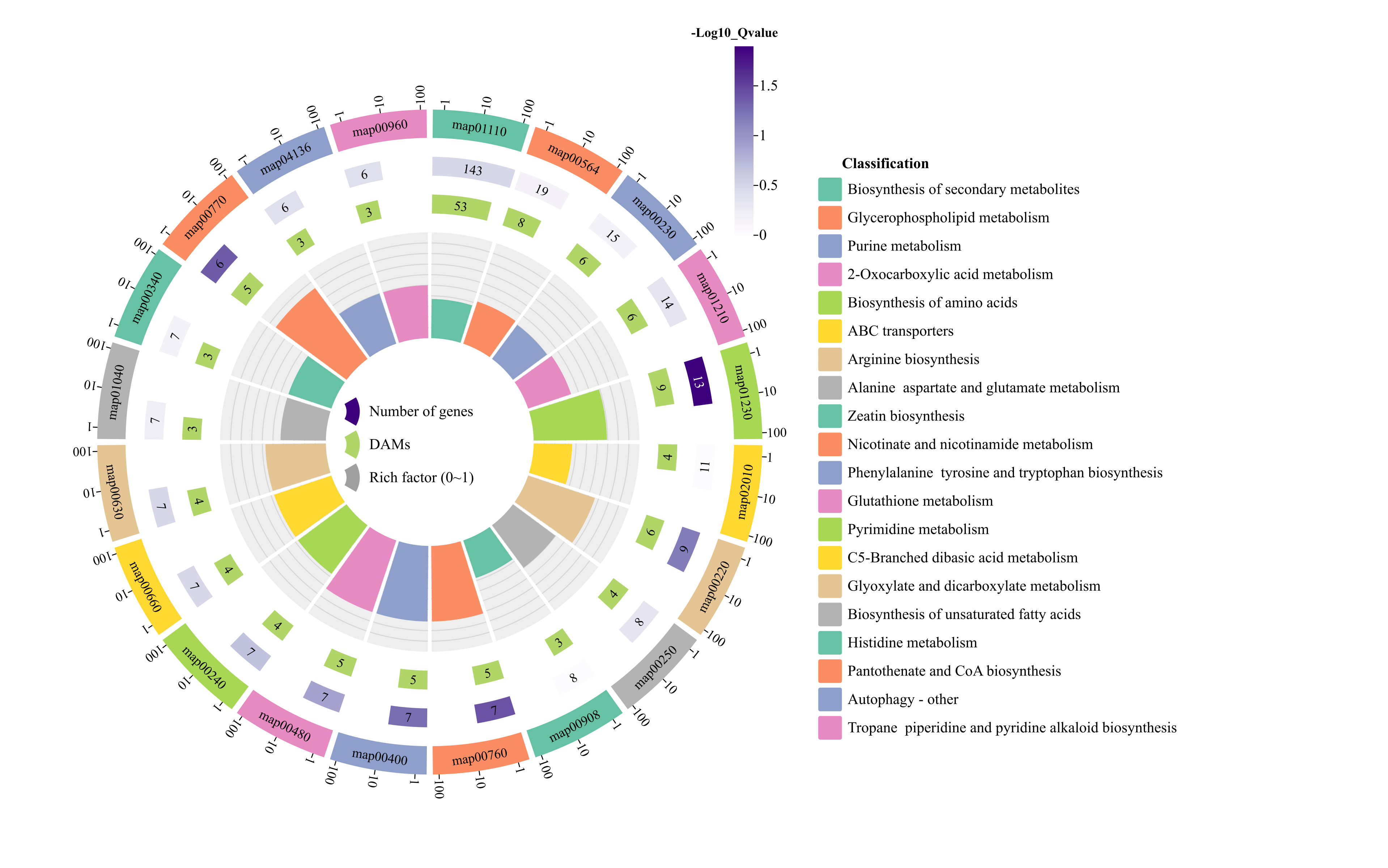

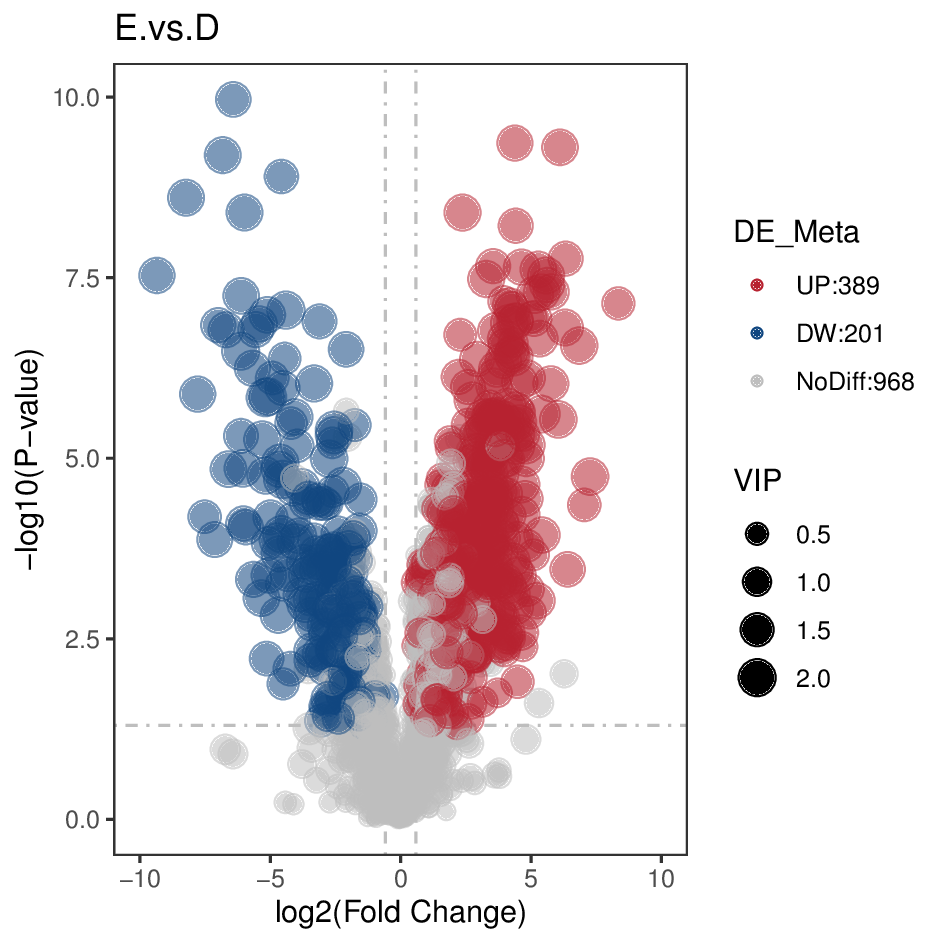


**R.vs.S Differential metabolite volcano map**

**Fig.S3** DAMs and DEGs in each control group.

**Fig.S4** Gene Function Classification (GO) and Kyoto Encyclopedia of Genes and Genomes (KEGG) Classification.


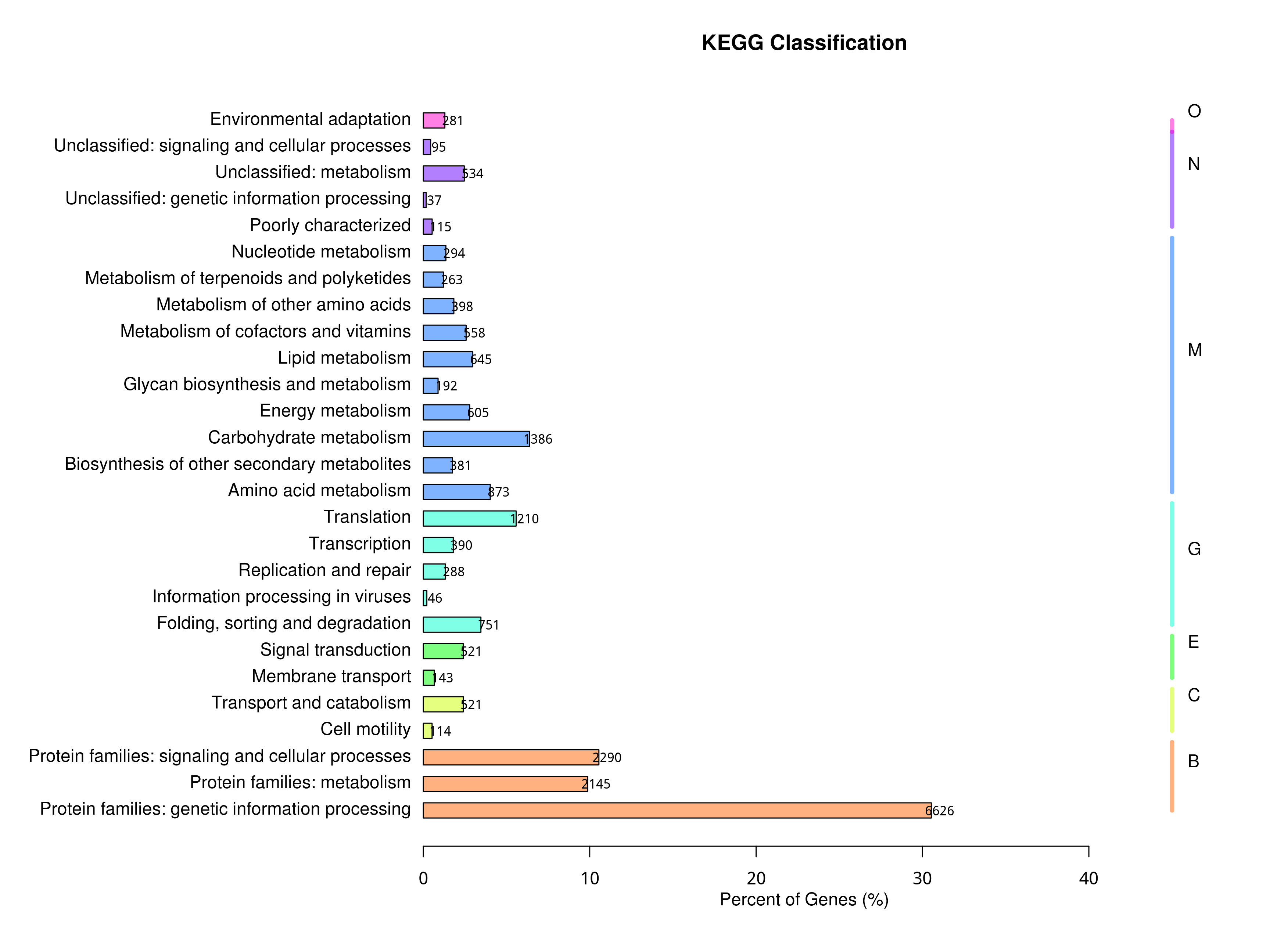

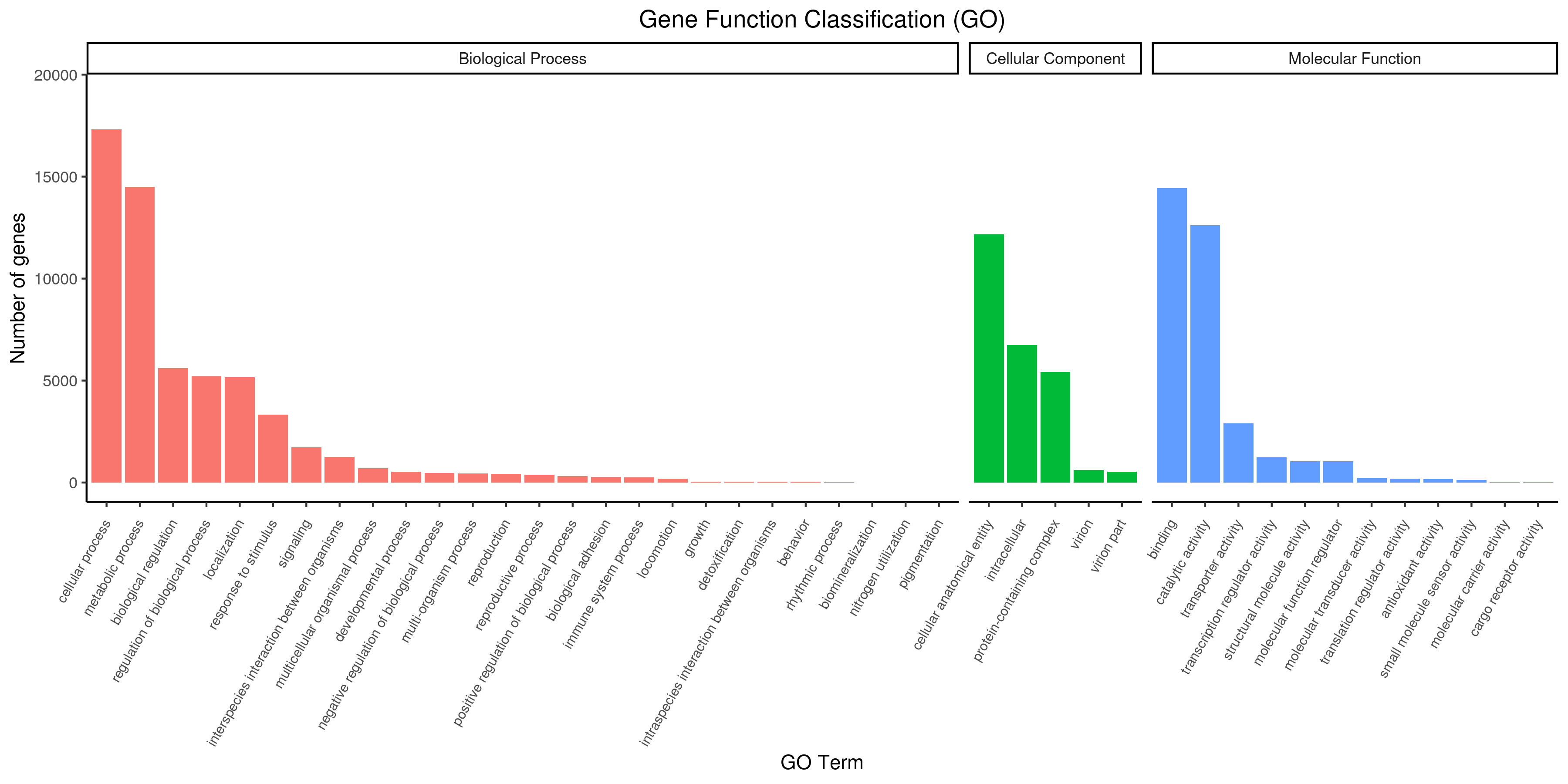

Supplement: Supplementary file 1 [file DataSheet1.docx]
